# Dodging blood brain barrier with “nano” warriors: Novel strategy against ischemic stroke

**DOI:** 10.7150/thno.64806

**Published:** 2022-01-01

**Authors:** Suhel Parvez, Medha Kaushik, Mubashshir Ali, Mohammad Mumtaz Alam, Javed Ali, Heena Tabassum, Pooja Kaushik

**Affiliations:** 1 Department of Toxicology, School of Chemical & Life Sciences, Jamia Hamdard, New Delhi 110062, India.; 2 Drug Design & Medicinal Chemistry Lab, Department of Pharmaceutical Chemistry, School of Pharmaceutical Education and Research, Jamia Hamdard, New Delhi, 110062, India.; 3 Department of Pharmaceutics, School of Pharmaceutical Education and Research, Jamia Hamdard, New Delhi - 110062, India.; 4 Division of Basic Medical Sciences, Indian Council of Medical Research, Ministry of Health and Family Welfare, Govt. of India, V. Ramalingaswami Bhawan, P.O. Box No. 4911, New Delhi 110029, India.

**Keywords:** Ischemic stroke, Nanomedicine, Drug delivery system, Intranasal drug delivery, Blood brain barrier, Neuroprotection

## Abstract

Ischemic stroke (IS) is one of the leading causes of death and disability resulting in inevitable burden globally. Ischemic injury initiates cascade of pathological events comprising energy dwindling, failure of ionic gradients, failure of blood brain barrier (BBB), vasogenic edema, calcium over accumulation, excitotoxicity, increased oxidative stress, mitochondrial dysfunction, inflammation and eventually cell death. In spite of such complexity of the disease, the only treatment approved by US Food and Drug Administration (FDA) is tissue plasminogen activator (t-PA). This therapy overcome blood deficiency in the brain along with side effects of reperfusion which are responsible for considerable tissue injury. Therefore, there is urgent need of novel therapeutic perspectives that can protect the integrity of BBB and salvageable brain tissue. Advancement in nanomedicine is empowering new approaches that are potent to improve the understanding and treatment of the IS. Herein, we focus nanomaterial mediated drug delivery systems (DDSs) and their role to bypass and cross BBB especially via intranasal drug delivery. The various nanocarriers used in DDSs are also discussed. In a nut shell, the objective is to provide an overview of use of nanomedicine in the diagnosis and treatment of IS to facilitate the research from benchtop to bedside.

## Introduction

Stroke is a medical condition that results in physical and psychological impairments and is also one of the major causes of mortality. The statistical data presented by World Health Organisation (WHO) suggest that approximately 15 million people encounter stroke annually at the global level. Clinically, stroke results in permanent disabilities that become a prodigious burden on the kinsfolk and community, which turns out to be around 61 million disability-adjusted life years (DALYs) in 2020, worldwide 1, 2. Stroke can be classified into three categories, ischemic stroke (IS), hemorrhagic stroke, and transient ischemic attack (TIA). Ischemic stroke occurs as a result of disruption of blood flow due to thrombotic and embolic events, which encompasses 80-90% of all stroke cases 3. Hemorrhagic stroke occurs with the bursting of blood vessels ensuing in blood leakage and TIA is considered short episodes of embolism of clots that temporarily obstruct the blood flow. The TIA-associated damage is minor compared to the other two conditions but can become a potentially irreversible and deadly risk for ischemic and hemorrhagic strokes 4, 5.

Till now, the first-line treatment approved for IS is tissue plasminogen activator (tPA). After fulfilling several selection criteria, it needs to be administered intravenously within 3 h to 4.5 h (for certain patients) of stroke attack. For patients with large vessel occlusion, endovascular thrombectomy is the standard treatment 6. The guidelines given by the American Heart Association (AHA) and American Stroke Association (ASA) for the early management of IS concerning endovascular treatment suggests that the related procedures must be achieved within 6 h after stroke commencement and are grounded on the outcome of 5 major clinical trials, namely, MR CLEAN (NCT0252361), ESCAPE (NCT02930018), EXTEND-IA (NCT03340493), SWIFT-PRIME (NCT01657461) and REVASCAT (NCT01692379) 1. This data reflects that currently, there is no active treatment for most of the patients with acute ischemic stroke. Therefore, the major emphasis of stroke research is to understand the pathological cellular and molecular mechanisms and to develop potent treatments that can alleviate brain damage from an ischemic insult.

The onset of cerebral ischemia initiates a complex cascade of pathological reactions in a sequential manner. To begin with, cessation of cerebral blood flow (CBF) leads to scarcity of oxygen and glucose, due to which the levels of adenosine triphosphate (ATP) get reduced, promoting rapid calcium influx that subsequently induces the important pathological events of ischemia, i.e., glutamate excitotoxicity, over production of reactive oxygen species (ROS), mitochondrial dysfunction, inflammation and apoptosis 7. These events result in irreversible tissue damage and infarction 8. Physiologically, ROS are indigenous by-products, majorly produced by oxidative phosphorylation (OXPHOS) in mitochondria, regulating various cell signaling pathways that further regulate different cell functions. An in-built antioxidant defense mechanism comprises various antioxidants like superoxide dismutase (SOD), glutathione (GSH), peroxidases, vitamins, coenzyme Q10 that proactively quench the ROS and maintain its level for proper maintenance of cell 9,10. The improper functioning of this defense mechanism leads to oxidative stress, leading to mitochondrial malfunctioning. Hence, an in-depth understanding of pathological changes in neuro microvasculature is required to develop an active pharmacological molecule or compound fighting against cerebral ischemia 11, 12. Therefore, neurotherapeutics that regulates mitochondrial oxidative stress by combating ROS production could be promising therapy for IS treatment.

Broadly, the two main approaches developed for treating IS are early recanalization or thrombolysis and neuroprotection. There are several thrombolytic drugs, and tPA has received Food and Drug Administration (FDA) approval. Still, due to its limited therapeutic time window, its use is restricted to a narrow range of patients. With the help of these thrombolytic drugs, blood flow is reinstated; however, a secondary cascade of events occurs because of the overproduction of ROS by mitochondria and inflammatory molecules in the ischemic area. So, the need for neuroprotectants comes into the picture to efficiently suppress the ischemic/reperfusion (I/R) injury 13, 14. As an effective therapeutic approach, mitochondrial neuroprotection intends to avert cell death in the infarcted area by targeting single or multiple deleterious events. Neuroprotection therapy is beneficial in increasing the time window in acute IS and rehabilitation of neurological function in the subsequent recovery period. There is vast preclinical literature that found neuroprotective agents to be efficacious for successfully targeting mitochondrial dysfunction to prevent IS injury, though not a single compound has proven effective clinically for various reasons. Still, most drugs fail to cross physiological barriers like the blood-brain barrier (BBB) 15. Therefore, innovative strategies are required with enhanced efficacy, delivery efficiency, and low side effects. In the past few years, nanoscience has gathered immense attention, as it provides safe and effective drug delivery systems or strategies (DDSs) that can even cross BBB. This is due to their better stability and active/passive directing features that improve drug concentration at the lesion area to attain necessary therapeutic effects 16, 17. A comprehensive understanding of the IS pathophysiology is required for the design and application of different nanotherapeutic strategies. Therefore, in this review, we briefly described the pathological events that occur upon ischemic injury with a major focus on mitochondrial damage and the current treatments available with their limitations. More significantly, this review provides an overview of nanoparticle (NPs) applications for IS management, primarily through the mitochondrial pathway. We have discussed the different DDSs and the nanocarriers involved in the DDSs, which might deliver direction and fruitful information for NPs use for translational research and provide medications from bench to bedside.

## Pathophysiological events involved in IS

**Figure 1:**
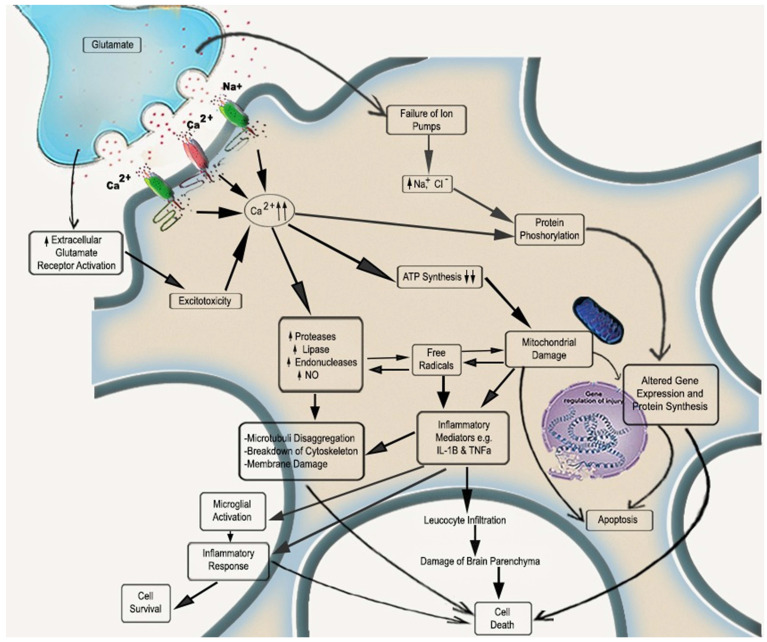
**Molecular mechanisms initiated during acute ischemic stroke.** With the disruption of cerebral blood availability, energy production decreases. In the scarcity of energy supply, the ion pumps fail along with the generation of free oxygen radicals, mitochondrial injury, leukocyte infiltration, and release of excitotoxins. Over accumulation of calcium leads to activation of phospholipases and proteases followed by membrane damage and cytoskeleton damage that ultimately leads to cell death. The inflammatory pathway may also be involved in cell survival by microglial activation.

In physiological conditions, the range of CBF is 50-60 mL/100 g tissue/min, which, when reduced to 20 mL/100 g tissue/min, leads to diminished neuronal functions and electrical silencing. An additional decrease in CBF will damage the neuronal cells irreversibly due to metabolic disablement 18. Furthermore, the diminished blood supply will decrease glucose, oxygen, and other vital nutrients in the brain. As a result, energy failure is followed by acidosis, imbalance in ion movements, and elevation in intracellular calcium. Subsequently, glutamate excitotoxicity, oxidative stress, inflammation, mitochondrial dysfunction, and apoptosis become key players in the progression of IS (Figure 1) 19.

### Glutamate induced excitotoxicity

Due to reduced oxygen and glucose supply, OXPHOS gets affected, resulting in decreased ATP production and demolition of the ion pump role. Under I/R injury, due to damaged mitochondria, energy gets produced anaerobically and almost entirely in need of anaerobic fermentation of glucose. The production of ATP by anaerobic metabolism is less than aerobic metabolism hinders the functions of ATP-dependent ion channels. ATP deficiency is also responsible for protein and lipid degradation in cell-matrix and destroys cell integrity 18, 20. Further brain damage occurs due to phospholipid degradation responsible for generating free fatty acids, arachidonic acid, etc. The Na^+^/K^+^-ATPase malfunction leads to neuronal depolarisation with an unusual influx of Na^+^ and efflux of K^+^. These depolarisations activate the voltage-gated calcium channels, which further allows rapid inflow of Ca^2+^, which results in the secretion of amino acids like glutamate 21. Excessive glutamate accumulation and overactivation of metabotropic glutamate receptors along with α-amino-3-hydroxy-5-methyl-4-isoxazolepropionic acid (AMPA) N-methyl-D-aspartate (NMDA) receptors take place, resulting in an imbalance of calcium homeostasis. To isolate these elevated Ca^2+^ levels, the energy reserves depleted very quickly 1, 22. Calcium overload is accountable for deleterious cascades through mitochondria-mediated oxidative mechanisms such as apoptosis 22, 23.

### Oxidative Stress

The downstream repercussion of excitotoxicity is oxidative stress due to over-production of ROS, which is well known to activate cell death by apoptotic and necrotic pathways, which decide resultant infarct size 24. Calcium overload, especially in mitochondria, excessively activates Ca^2+^ dependent enzymes like proteases, phospholipases, and endonucleases, along with calmodulin-dependent enzymes like nitric oxide (NO) synthase. Due to Ca^2+^ mediated impairment of mitochondria and ROS defense mechanisms, superoxide anions aggregate in the cytosol as superoxide dismutase also become overwhelmed by overproduction of ROS 25, 26. Upon reperfusion, such mechanisms charged up with oxygen supply. Furthermore, ROS can directly or indirectly affect various cell macromolecules by regulating signal transduction pathways such as lipid peroxidation (LPO), protein oxidation, and DNA fragmentation. This overproduced ROS also affects the BBB and makes it leaky by altering the expression and molecular organization of BBB 27, 28.

### Inflammation

Inflammation is the immediate response after vessel occlusion and is involved in all stages of IS 29. Within minutes, damage-associated molecular patterns (DAMPs) and cytokines released from the ischemic region can stimulate pattern recognition receptors (PRRs) on microglia and astrocytes. PRRs help cells to detect DAMPs by activation of toll-like receptors (TLRs) and inflammasome 30, 31. TLRs activate transcriptional mediators, regulated by nuclear gene expression, which release pro-inflammatory factors. Activation of inflammasome involves activation of caspase cascade, which cleaves inactive pro-inflammatory cytokines IL-1β and IL-18 into active cytokines for release. Within few hours of the ischemic attack, microglial activation takes place 32, 33. PRRs activation on microglia leads to transcriptional stimulation of NF-κB expression and other cytokines like TNF-α, IL-1β, IL-6, IL-18, and NO, which activate inflammatory cascade in endothelial cells and astrocytes, via chemokines and cytokines production 34. Consequently, leukocyte infiltration occurs in the injured region and expresses antigens between dendritic and T cells. At the time of ischemic insult, activated astrocytes secrete various pro and anti-inflammatory cytokines such as IL-1α, IL-1β, and chemokines like monocyte chemotactic protein-1 (MCP-1), ultimately resulting in excessive oxidative stress and matrix metalloproteinases (MMPs) activation 31. The activation of MMPs affects the basal lamina of the extracellular matrix and, therefore, directly causes the breakdown of BBB, leading to additional inflammatory insults. In chronic stages, the innate immune system transforms to an anti-inflammatory M2 phenotype. It shows repair-oriented features by secreting growth factors and anti-inflammatory cytokines like IL-4 and IL-13, inhibiting inflammation 35, 36. Phagocytosis is also involved in resolving post-ischemic inflammation by clearing cellular debris 37.

### Mitochondrial dysfunction and Apoptosis

Mitochondria comprise about 25% of neuronal cell volume and generate most of the energy by OXPHOS 38. They have an essential role in cell viability and are prone to injuries that result in necrotic and apoptotic cell death. Mitochondria involve the intrinsic pathway of apoptosis 39. After IS, disruption of OXPHOS produces a large amount of ROS, leading to decreased ATP production, the collapse of mitochondrial membrane potential, mitochondrial swelling, and ultimately opening of mitochondrial permeability transition pore (mPTP) 40. The transient opening of mPTP helps eliminate toxic levels of ROS, but the permanent opening is associated with Ca^2+^ release and various apoptotic proteins like cytochrome c and apoptosis-inducing factor (AIF). There is a vast literature that suggests that group of anti-apoptotic (Bcl-2, Bcl-xL, Bcl-w) and pro-apoptotic proteins (Bax, Bak, Bim, Bad, Noxa, and p53) of the B-cell lymphoma (BCL-2) family are key regulators of apoptosis. On ischemic injury, the level of pro-apoptotic proteins is raised, and the anti-apoptotic becomes diminished. Bax translocates to mitochondria and facilitates cytochrome c from mitochondria into the cytoplasm 41, 42. Once in the cytoplasm, cytochrome c activates caspase cascade, leading to apoptotic cell death 43.

Therefore, for the normal functioning of a cell, it is necessary to maintain low levels of ROS. On the other hand, the augmented elevation of mitochondrial activity comprises of intrinsic risk of elevated ROS levels and inflammation. In the pathological conditions of cerebral ischemia, the equilibrium in the middle of ROS production and its clearance is hampered, resulting in oxidative stress-mediated signaling and cell injury 1.

## Strategies available for the treatment of IS

IS therapy can be classified as preventive therapy (pre-symptomatic), checking progression (symptomatic), and facilitating recovery (recovery stages) 44. Till now, the strategies available for the treatment of IS include thrombolysis, endovascular thrombectomy, anti-coagulant, anti-platelet therapy, along neuroprotective therapy [Table 1]. The first (thrombolysis and endovascular thrombectomy) focuses on eradicating or diminishing clots through medicines or mechanical surgeries. Moreover, the remaining therapies focus on minimizing deleterious ischemic damage to neuronal cells. Along with these strategies, nursing care of IS patients post-treatment is also of great value. It can prevent recurrence of clot formation by regulatory risk factors like obesity, high blood pressure, smoking, and diabetes mellitus 45, 46.

### Thrombolytic Therapy

Physiologically, plasminogen initiates a fibrinolytic process that breakdown to form plasmin in the presence of plasminogen activator. Plasmin is responsible for the degradation of solid fibrin into soluble products. When there is an imbalance between coagulation and fibrinolysis, several complications like cardiac arrest or cerebral stroke may occur 47, 48.

Until recently, the only treatment for IS approved by US FDA was intravenous administration of recombinant tPA (rt-PA or Alteplase) 49. tPA is the fibrin-specific mediator that triggers fibrin-bound plasminogen. There are clinical trials that have shown good outcomes with Alteplase. For example, the National Institute of Neurological Disorders and Stroke (NINDS) tPA trial demonstrated good results at 0.9 mg/kg Alteplase when given intravenously for 3 months compared to placebo and get licensed with 3 h time window from stroke onset 50, 51. On the other hand, the European Cooperative Acute Stroke Study II (ECASS II) and the Alteplase Thrombolytic for Acute Noninterventional Therapy in Ischemic Stroke (ATLANTIS-B) trials showed no expected outcome. Still, they were combined in a meta-analysis involving over 2000 patients treated within 6 h from stroke onset 52. The Alteplase showed a neutral effect at 4.5 h. The ECASS III trial validated the sustained advantage of Alteplase in 3-4.5 h, but delay after this time window increased the risk of thrombolytic mediated symptomatic intracerebral hemorrhage 53. In recent years, many new agents used as thrombolytics have been established both in the fibrin-specific and non-specific category, which fails to differentiate between fibrin-bound or free plasminogen 54, 55.

### Mechanical Thrombectomy

Endovascular mechanical thrombectomy (EMT) is another treatment for eliminating clots as additional to thrombolysis or subjects not eligible Alteplase IV 56. Various randomized multi-center studies in 2015 reflected that mechanical thrombectomy has several advantages over the use of intravenous rt-PA to treat intracranial vessel occlusion 57, 58. Therefore, EMT along with rt-PA can turn out to be a guideline-recommended therapy for IS with large vessel blockage. However, although this treatment attains absolute value, few patients attain this due to less popularity rate or delay in treatment 59, 60.

### Anti-Coagulant and Anti-platelet Therapy

IS can be prevented by anti-coagulant and anti-platelet therapy, with or without surgical procedures 61. Aspirin is widely used as an antiplatelet drug. This inhibits platelet adhesion and circumvent recurrent thrombolysis and thereby prevents clot formation 62. Evidence suggests that dual therapy, i.e., clot dissolution and anti-platelet agents caters better results than monotherapy. However, this dual therapy is associated with bleeding as its adverse effect 63. If given without a proper selection of patients, anti-coagulants lead to symptomatic intracranial hemorrhage that fails to show the expected benefit. The common anti-coagulants used are heparin, rivaroxaban, danaparoid, warfarin, apixaban, dabigatran 64. The peculiarity of the time window related to anti-coagulants for secondary damage of IS is still debatable. Exact circumstances for deliberation of early anti-coagulation possibly will include extracranial arterial dissection or the occurrence of severe cardiac thrombus 65, 66.

### Neuroprotective Therapy

The presently available treatment strategy for I/R injury is only symptomatic and rarely targets the fundamental cause of the disease. Hence, new therapies are required to suppress destructive molecular events and neuronal death. Neuroprotection is a promising therapeutic approach that aims to extend neuronal survival after IS to extend the therapeutic window and involves neurological repair and recovery of the functional outcomes 67. However, currently, there is no effective therapy that can avert neuronal tissue damage following IS.

Neuroprotective therapy specifically arrests the pathological events involved in IS 13, 68. Neuroinflammation is well known for its harmful effects, thereby promoting irreversible cell death by generating ROS and pro-inflammatory cytokines. Several investigations have reported the involvement of mitochondrial dysfunction in IS 1, 69. As described previously, ROS leads to mitochondrial dysfunction, which plays a central role in the pathogenesis of IS 70. In such conditions, strategies that specifically target mitochondria and improve mitochondrial bioenergetics will promote extraordinary therapeutic potential, presenting an intrinsic antioxidant power 71. The neuroprotective agents that scavenge ROS can also target mitochondrial dysfunction, thus shielding the ischemic neurons from irreversible damage 72. Based on their activity, neuroprotective agents can be categorized. Though their therapeutic efficacy, when used alone, did not show encouraging results, they are generally used in amalgamation with other drugs. The classes of neuroprotectants include ROS scavengers, calcium channel blockers, GABA agonists, NO antagonists, calcium chelators 73.

### Shortcomings of current therapies

Systemic administration of thrombolytic agents may cause hemorrhagic complications due to the activation of plasminogen in non-ischemic regions. Therefore, site-specific delivery is required for the effective treatment of IS 74. In addition, chemotherapy for IS has one more challenge: to evade physiological barriers present in the brain. A neurotherapeutic drug has to traverse three barriers for the treatment of IS: BBB, arachnoid epithelial layer, and blood-cerebrospinal fluid (CSF) barrier. Hence, the next generation neurotherapeutics are developed with the expectation that they can cross the barriers and reach the ischemic site efficiently 75, 76.

Within the past two decades, thousands of neuroprotective agents emerged, and nearly about hundreds progressed towards clinical trials. However, unluckily, not a single agent reflected unambiguous profits that satisfy the regulatory qualifications for approval in clinical trials. Nevertheless, most of them have proven to be effective preclinically 77. The inadequate methodological thoroughness of few preclinical studies and their failure to mimic clinical conditions completely contributed to the disappointments of clinical transformation. BBB plays a crucial role in homeostasis, and its specific permeability feature is also a major hurdle as it restricts the entrance of most of the neuroprotectants to the brain 78. As a result, BBB gets partially disrupted in ischemic conditions. Still, the level of leakage might not be satisfactory for delivering a particular quantity of drugs for effective IS treatment. Therefore, approaches to increase neuroprotectants uptake in the ischemic region will significantly upgrade the therapeutic efficiency associated with their clinical translation 79.

The reasons for the clinical failure of neuroprotectants are as follows: firstly, the IS treatment that takes place in the clinical setup is generally started beyond the proposed therapeutic window of the drug. Secondly, many neuroprotectants cannot cross through BBB. Likewise, the drugs envisioned to target mitochondria failed to get away as mitochondria is a bilayer structure with high negative potential and are extremely impermeable compared to the plasma membrane. Therefore, targeting neurotherapeutics to mitochondria has become challenging due to the mitochondrial membrane's highly selective and impermeable feature. Third, the co-morbidities like diabetes mellitus, dementia, aging, hypertension significantly reduce the neuroprotectant efficiency. Fourth, I/R injury produces heterogeneity in location and intensity, which requires different drugs at different doses. Fifth, clinically, it is tremendously difficult to analyze and categorize patients in batches and choose a drug's effect 79.

## Blood-brain barrier and application of NPs for neuroprotection in IS

The brain is the most fragile and complex organ in the human body. It has a series of barriers to protecting from invading pathogens, xenobiotics, and neurotoxic molecules in blood circulation. These barriers, the blood- CSF barrier, BBB, blood-retinal barrier, and blood-spinal cord barrier, have a different level of permeability 80. Among all, BBB is the most widespread and selective. It is majorly composed of endothelial cells which are tightly connected and an intermittent layer of pericytes. The BBB integrity, endothelial transport of cells, along with an angiogenic feature to permit revascularisation, has been maintained by cerebral endothelium. For promoting the intactness of BBB, endothelial cells express certain proteins, specifically tight junction (TJ) and adherence junction (AJ). The transmembrane and cytoplasmic proteins constitute TJ, which include claudins, junction adhesion molecules (JAM), occluding, zonula occludens (ZO), along accessory proteins 81. However, BBB has a robust, cohesive system. It allows the selective passage of cells and small molecules to the brain. With the help of the paracellular mechanism of endothelial cells, ions and solutes movement takes place depending on the concentration gradient. The passage through endothelial cells is called transcellular, and equilibrium between transcellular and paracellular transport is pivotal to describe the extent of permeability in a healthy BBB. The passive diffusion of lipophilic molecules occurs via a transcellular pathway mediated by specific receptors to conveyance molecules like carbon dioxide. The passage of hydrophilic molecules like proteins and peptides depends on a special type of transport channels such as glucose transport-1 (GLUT-1) for the transportation of glucose 82. Additionally, transport can also occur by the formation of cellular invaginations called caveolae. Transcytosis is presently studied as an effective method for transporting therapeutic drugs in the brain 83.

BBB opens briefly, which can last from several minutes to hours during an ischemic stroke. A refractory interval follows up, and then the BBB opens again for a prolonged period, lasting from several hours to days. Reperfusion is essential to prevent further cerebral injury; however, this can lead to aggravated damage, thereby correctly calling it reperfusion injury. BBB reopens after the refractory interval is caused by this, leading to endothelium activation, ROS production, leukocyte recruitment, cytokine production, and edema formation 16, 76.

Loss and disruption of TJ primarily cause the BBB dysfunction developed during an ischemic stroke. Matrix metalloproteinases (MMP), which play a larger role in tissue remodeling, also degrade TJ. This class of metalloproteinases also degrades BBB extracellular matrix (type IV collagen), increasing BBB permeability. A correlation is found between the higher levels of MMP-9 and increased BBB permeability and the occurrence of severe disease in stroke patients and stroke animal models. During the ischemia-reperfusion, there is a heightened level of nitric oxide (NO), which activates MMP-9 and MMP-2 and therefore enhancing the permeability of BBB. Vascular endothelial growth factor (VEGF), which is an angiogenic factor, also promotes MMP activity. A decreased transepithelial electrical resistance and claudin-5 and occluding expression are found when endothelial cells are treated with VEGF. The *in vivo* inhibitors of VEGF caused the reduction in BBB permeability and infract volume in hypoxia models 76,126.

The inflammatory response further promotes BBB breakdown and cell death on stroke through the activation of microglial cells and infiltration and activation of peripheral leukocytes. Brain's first line of defense, which is microglia, gets activated and starts to release nitric oxide, which in turn produces ROS, cytokines (for example. tumor necrosis factor-alpha (TNF)-α, interleukin-1beta (IL-1β) and IL-6), and chemokines (for example, macrophage inflammatory proteins-1alpha (MIP-1α)/CCL3, monocyte chemoattractant protein-1 (MCP-1)/CCL-2 and chemokine (C-X-C motif) ligand-1 (CXCL-1)). These inflammatory modulators excite the endothelial cells and start the nuclear factor (NF)-kB pathway, stimulating the expression of adhesion molecules [P-selectin, vascular cell adhesion protein (VCAM), and intercellular adhesion molecule-1 (ICAM-1)]. This series of actions conclude recruiting brain parenchyma and invading the peripheral leukocytes, further promoting and continuing the inflammatory cascade 16, 126.

When delivering a drug in the case of a stroke, events such as compromised TJ and an early and a delayed opening of BBB should be considered. Increased BBB permeability and/or the expression of few receptors on the luminal side of endothelial cells may improve the chance to increase the rates of NP bypassing the BBB. The BBB itself is a good target for improving drug delivery in the ischemic brain 76.

Therefore, in-depth knowledge of BBB is required to foster new strategies for delivering neuroprotective and regenerative molecules into the brain. To become an effective neurotherapeutic, drugs must have efficient and precise brain delivery, following IS. The incapability of drugs to cross the BBB underlines the requirement for developing NPs based strategies for drug delivery 84. NPs display aids in averting drug degradation, improved pharmacokinetic profiles, and access to the neurovascular unit. These nano formulations pass through the BBB and make their way to neurons through different routes. NPs penetrate the brain through the TJ of endothelial cells and enable the drug to pass through BBB 85. NPs travel via endocytosis and transcytosis, facilitating the drug transfer to the targeted ischemic region or penumbral region (the region surrounding the ischemic site which can be salvageable). BBB comprises several receptors also that allow ligands to bind and internalize into the cells. Therefore, the ligand-based transport through BBB could be accomplished by the intervention of NPs. By the interface between receptors and ligands, the receptor facilitated transport has become the most effective strategy to deliver NPs to the brain crossing through BBB 86. However, these nanotherapeutics are successful in preclinical studies and are yet to be implemented clinically. In the later sections, we summarised several promising DDSs and nanocarriers for the management of IS [Table 2].

Similarly, mitochondrial insult and related oxidative stress have been influential factors in the pathology of IS. Presently, several neuroprotective therapeutics are targeting mitochondria for the treatment of IS. Keeping in view, several drugs are being modified into a nanoformulation to act as an antioxidant and target mitochondrial oxidative stress. Several studies are described in the following section related to antioxidant NPs and their therapeutic target as mitochondria and related oxidative stress.

## Antioxidant NPs targeting mitochondrial oxidative stress

There are NPs that have redox property, and because of which, they can be used as possible biologically active antioxidants. Over the last few years, extensive research has emphasized nanomaterials can simulate antioxidant properties to inhibit apoptosis and promote cell survival after IS. However, a few NPs extensively studied for their antioxidant properties are Cerium (CeO2, Ceria) NPs, Platinum NPs, Selenium NPs, and Gold NPs [Table 3].

As previously described, the production of ROS as a result of excitotoxicity in ischemic stroke is a major cause of necrosis, apoptosis, and macrophagic pathway activation, further determining the final infarct size. Subsequently, it has also been established that mitochondrial injury post-ischemia results in excessive ROS production in the ischemic penumbra, further leading to neuronal damage. Recent therapeutic advancements related to IS are also directed towards intracellular pathways for better neuroprotective effects. Several studies have explored the neuroprotective effects of mitochondrial-targeted drugs encapsulated in the nanocarriers 87, 88. There are several mitochondria-targeted nanocarriers, namely, triphenylphosphonium (TPP), dequalinium (DQA), peptide-based nanocarriers, Liposomes, Transition Metal complexes, and polymeric nanocarriers 87, 89, 17, 90. Additionally, ROS scavenging effects of these nano formulations have also been explored in these studies. It has been found that mitochondria-targeted nanoparticles have anti-oxidative effects in severe neuroinflammatory conditions, such as observed in IS 91, 92.

Transition metals-based nanocarriers encapsulating mitochondrial-targeted drugs are most effective in scavenging the ROS produced from mitochondrial insults. Recent studies have exhibited the antioxidative properties of cerium oxide (CeO_2_)/ceria nanoparticles, as they are found to mimic catalase and SOD activity due to their +3 and +4 oxidation states 89, 17, 93. Modified ceria nanoparticles have also been designed recently for their enhanced neuroprotective and therapeutic effects. *Bao et al.* (2018) developed Angiopep-2 and polyethylene glycol coated cerium oxide nanoparticles loaded with Edaravone for effective stroke treatment, consequently found to have ROS elimination effects in stroke conditions 94. In a similar study, *Arya et al.* (2016) established the efficient localization of polyethylene glycol coated ceria nanoparticles in rodent brains resulting in a significant reduction of oxidative stress 95. Another study by *Estevez et al.* (2019) demonstrated that stabilization of ceria nanoparticles with different ratios of citric acid and EDTA have greater neuroprotective and antioxidative activity in ischemic mouse brain slices 96. Similarly, enhanced mitochondrial ROS scavenging effect of ceria nanoparticles by conjugation with TPP ions has also been reported 89. Apart from cerium oxide, few other nanoparticle formulations have been identified to mimic SOD activity, mesoporous silica, iron oxide, platinum, and selenium, thus expressing strong antioxidative and anti-inflammatory effects 17, 97, 98.

Furthermore, several polymeric nanoparticles have also been developed recently for mitochondrial targeting. Polymer-based nanocarriers such as polyanhydrides, chitosan nanoparticles with surface modification by TPP, and polyethylene glycol nanocarriers are extremely effective when loaded with free radical scavengers 89. A similar study demonstrated enhanced neuroprotective effects of Gallic Acid-loaded chitosan nanoparticles among *in vitro* and *in vivo* models of IS when compared with Gallic Acid administration 99. *Sun et al.* (2019), in their study, found the amelioration of mitochondrial ROS by application of resveratrol (a mitochondrial function enhancer drug) loaded solid-lipid nanoparticles, thus establishing its antioxidative effect 100. Neuroprotective effects of curcumin are being studied lately. However, studies have found that curcumin-loaded nanoparticles such as DQAsomes, poly(lactic-co-glycolic acid)-poly(ethylene glycol) (PLGA-PEG), and solid-lipid nanoparticles have greater ROS scavenging and neuroprotective effects as compared with direct curcumin application 101, 102, 90. In light of the same experiment, *Ghosh et al.* (2010) developed nano-capsulated Quercetin, a naturally occurring flavonoid with immense free radical scavenging properties. They found a significant increase in the neuroprotection against mitochondrial oxidative damage among young and old rats by nano-capsulated Quercetin compared with Quercetin treatment 103, 104. Another study by *Li et al.* (2020) established the enhanced neuroprotective and ROS-responsive activity of dendrimer nanoparticles conjugated with Salvianic acid A, an effective antioxidant in IS 105. Multi-antioxidative effects of melanin nanoparticles for better antioxidative therapy in IS have also been explored by *Liu et al.* (2017). The study found the attenuating effects of melanin nanoparticles on inflammatory responses by suppressing inflammatory mediators and cytokines 106. Moreover, fullerene (C_60_) nanoparticles have also been identified as the potent free radicals' scavenger and protecting neuronal injury from reperfusion 107.

In addition to antioxidative drugs, several new components such as inflammatory cytokines and antioxidative enzymes loaded nanoparticles are also being screened for their free radical scavenging and neuroprotective properties 98. *Xu et al.* (2017) provided strong evidence in their study that tumor necrosis factor (TNF)-α-loaded poly (ethylene glycol)-b-(poly(ethylenediamine-glutamate)-g-poly(l-lysine)) (TNF-α/PEG-b-(PELG-g-PLL)) nanoparticles exhibit strong therapeutic activity in ischemia/reperfusion injury. It was found that TNF- α attenuated the oxidative stress and inflammatory response post-reperfusion injury 108. Another study demonstrated the development of PLGA and liposomes nanoparticles loaded with activated SOD enzyme in the *in vivo* model of cerebral ischemia, exhibiting neuroprotection against reperfusion injury and reduced inflammatory response 109. In another development, studies have also been published indicating the use of radical-containing nanoparticles (RNP). The treatment with RNPs has been found to have suppressive effects on lipid peroxidation and protein oxidation along with scavenging effects against OH, ROO, and O_2_^-^ species, thus highlighting their neuroprotective capacity 110, 111. *Saralkar et al.* (2020), in their study, optimized a PLGA nanoparticle formulation for the mitochondrial outer membrane protein mitoNEET ligand NL-1 and found that this formulation displayed strong protection against peroxide generation in the *in vitro* ischemic model 112. Additionally, artificial nanoparticles with multienzyme activity, named, nanozymes are also developed, displaying neuroprotection by scavenging reactive oxygen and nitrogen species. *Zhang et al.* (2019) designed and developed hollow Prussian blue nanozymes (HPBN). The study found that HPBN attenuated apoptosis and oxidative stress, thus providing strong neuroprotection against ischemic insults 113.

Identification of a therapeutic target often solves half of the problem in drug development and treatment of IS; however, a method to deliver the drug successfully to its target is another major challenge. Several drug delivery strategies are available for targeting brain cells and organelles. Detailed discussion is provided regarding different drug delivery systems available to target the brain and their merits and demerits.

## Drug delivery strategies for targeting brain

**Figure 2:**
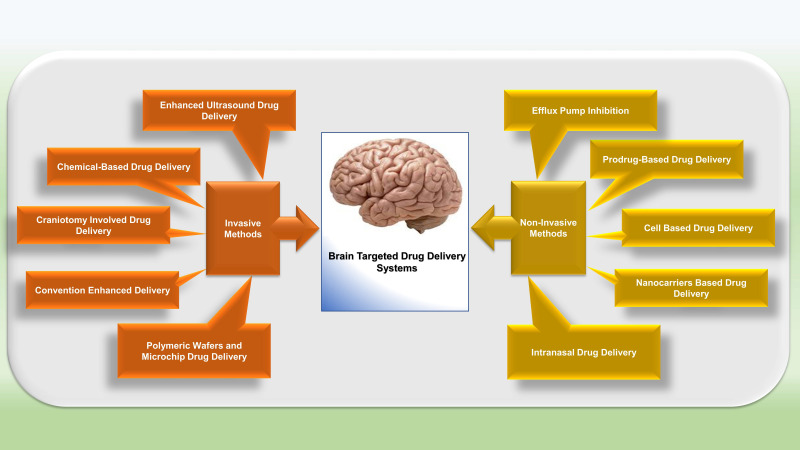
**Drug delivery systems used for targeting the brain.** The two major drug delivery strategies are invasive and non- invasive.

Several DDSs have been studied that were able to disrupt or overcome BBB and facilitate the transport of drug molecules to the CNS. Broadly these strategies can be classified into three categories: invasive, non-invasive, and other techniques (Figure 2) 78.

### Invasive Strategies

#### Chemical-based drug delivery

Many invasive strategies are employed to hamper BBB and increase drug delivery to the brain. One of the invasive techniques is Osmotic disruption of BBB, which temporarily involves shrinkage of endothelial cells, opening TJs, and seepage of the drug to CNS 82, 114. However, this technique has few drawbacks: it mediates the transport of plasma protein to the brain, glucose uptake gets disturbed, and micro-embolism takes place with neurotoxicity, which disturbs brain functions. The two main vasoactive agents used are bradykinin and histamine that disrupt BBB and facilitate drugs transportation. Bradykinin activates Bradykinin 2 receptors (B2Rs), modulates caveolin-1 and caveolin-2, and increases permeability by the involvement of potassium (KATP) channels 115.

#### Enhanced ultrasound drug delivery

Usage of ultrasound waves is one of the versatile approaches to reversibly and temporarily opening BBB to enhance drug transportation to the brain. In this technique, microbubbles (MBs) are used as contrast mediators. The diameter of the MBs used in this technique is 1-10 μm and consists of semi-firm lipid and albumin shells coated with perfluorocarbon 116. Systemically, these bubbles will be administered. The principle of acoustic energy is utilized to apply pressure on endothelial cells and open TJs, which subsequently increases the BBB permeability. MBs are teamed up with low-intensity Focus Ultrasound (FUS), and this system is collectively known as MB enabled FUS (MB-FUS). This system is successfully used with other DDSs for improved targeted delivery of drugs to the brain 117.

#### Drug delivery involving craniotomy

This is the direct method for targeting the specific region of the brain without hindering the peripheral organs through intracerebral/ intraventricular injection. In intraventricular delivery, a catheter is used to implant a drug reservoir into the scalp, providing controlled drug discharge to the specific ventricle site. This system follows a diffusion mechanism and facilitates the steady dispersal of drugs inside the brain. In this system, large doses of drugs are required to achieve a significant therapeutic response, as diffusion declines with an increase in the distance 115.

#### Convention enhanced delivery

This is the conventional method and utilizes the unceasing infusion method and pressure gradient to dispense a large volume of drugs at the targeted site through intracranial catheter, thereby overcoming the disadvantage of the intracerebral system of drug delivery. This method has few shortcomings as it provides drug exposure to surrounding tissues, failed to optimize the formulations, less stability of the drug in the target area 118.

#### Strategies involving polymeric wafers and microchip for drug delivery

For circumventing the BBB, polymer nanotechnology emerged as an advanced approach for drug delivery. Wafers based on polyanhydride were used to distribute the drug at the target site of the brain. Programmable microchips are used intracranially for the controlled release of the drug. There are two types of chips, namely active chip and passive chip. Active microchips are based on microelectromechanical systems (MEMS) and provide highly programmable drug delivery. In passive microchips, the drug is released by slow degradation of polymeric film surrounding the micro-reservoir. Delivery of multiple drugs can also be possible with the help of these chips 119.

### Non-invasive Strategies

Non-invasive approaches are based on the endogenous mechanisms for drug transportation through BBB. These strategies comprise prodrug strategy, inhibition of efflux pump, the alternative route of administration, and nanocarriers (described separately in later sections) based drug delivery.

#### Pro-drug Strategy

For better BBB permeation of a drug, the lipophilic characteristics must be enhanced. Prodrug approach facilitates chemical modification of drug molecules to change their lipophilic behavior, water solubility and increase permeability. Prodrugs have chemical entities and parent drugs to make it active moiety designed to target a particular site. Prodrug strategy is exploited for the transport of neurotherapeutics to treat IS. This approach is also followed by mitochondria targeting neurotherapeutics 115.

#### Inhibition of Efflux Pump

The presence of an efflux pump is another hurdle for the efficient transportation of drugs in the BBB. P-glycoprotein (P-gp) is found on the apical membrane of endothelial cells of BBB, which exerts efflux and results in poor availability at targeted brain tissues. Additionally, multidrug resistance-associated protein (MRP) present on endothelial cells is also associated with the efflux of cationic molecules. Physiologically, the role of P-gp and MRP is to protect the brain from xenobiotics or any other toxic substances that resultantly restrict the drug entrance to the brain. P-gp and MRP efflux inhibition are useful for delivering potent drugs to the brain 120.

#### Cell-based Therapy

This approach utilizes macrophages and types of stem cells as carriers for drug delivery and comes up as an effective approach for effective delivery. Paracellular and transcellular mechanisms are used to migrate macrophages to the brain 115, 121.

### Other developments in DDSs for targeting brain

#### Antibodies based drug delivery

Antibody-mediated therapy is also used to transport drugs; however, BBB restricts the entry of antibodies limits its use as significant DDSs for IS 122.

#### Mfsd2a- mediated drug delivery

This approach facilitates lysophophatidyl-choline (LPS) derivative mediated transportation as Mfsd2a, which is present on endothelial cells, restricts molecule transmission through transcytosis. This is considered a novel approach to target the brain 123.

## Nanocarriers in drug delivery system

Nanocarriers are also considered nano-vehicles as they facilitate the targeted and control release delivery of therapeutic ingredients in various diseases, including neurological disorders 124. The ability of nanocarriers to improve the permeation of drugs across BBB has added great value in DDSs. In the last few decades, the research for brain targeted delivery has been focused on polymeric NPs grounded on polylactic acid (PLA), poly (D.L-lactide-co-glycolide) (PLGA), and polyglycolic acid (PGA). To further improve the stealth and efficiency of polymeric NPs, the surface coating of NPs with PEG, chitosan, lectin, and D-α-tocopherol polyethylene glycol 1000 succinate (TPGS) can be helpful. Additionally to polymeric NPs, another approach for efficient brain targeting includes solid lipids NPs (SLNs). The importance of the nanocarriers has been proved by liposome-mediated delivery of IS drugs, opioid peptides, and anti-tumor agents 125.

### Intersecting BBB

The main strategies to overcome BBB are:

1. An increase in the transient permeability provokes the BBB paracellular pathway. Ultrasound/microbubbles and osmotic pressures are used to disrupt the tight junctions between adjacent endothelial cells. These two improve the BBB permeability causing an increase in the entry of the nanoparticles. There are few risks involved with this technique as it allows a direct entry of several compounds into the brain because of the loss of the homeostatic function of BBB and may cause cerebral toxicity.
2. There are two different mechanisms of transcytosis pathways.
3. Adsorptive-mediated transcytosis (AMT): Here, the binding of the nanoparticle and its cargo to the luminal plasma membrane of endothelial cells is enabled by the surface properties of the nanoparticles.
4. Receptor-mediated transcytosis (RMT): Here, endocytosis is promoted by the nanoparticles that contain different ligands on their surface that bind to specific receptors.
5. Adsorptive transcytosis is a complex process that involves several components like receptors and different signaling pathways. Two main routes are:
6. Clathrin-mediated endocytosis: This mechanism occurs in clathrin-enriched areas of the cell membrane. Clathrin-coated vesicles merge, leading to the formation of an early endosome, subsequently progressing to late endosomes and eventually merging with lysosomes leading to the degradation of their cargo.
7. Caveolin-mediated endocytosis: This mechanism occurs in lipid rafts where invaginations of the plasma membrane of approximately 80nm in size are found. Caveolin vesicles merge with other vesicles forming caveosomes. Their final fate depends upon the type of the cells.

For example, PGLA coated Diphtheria toxin (CRM197) successfully crossed the BBB by RMT and upregulated carrier-mediated transport (Caveolin-1-mediated transport). Further investigations have demonstrated that glycopeptide g7-NPs and CRM197 NPs reached different brain regions without disturbing the BBB integrity. Likewise, the phase -II clinical trials of PLGA NPs loaded with doxorubicin (NanoBB-1-Dox) revealed that it crosses the BBB more efficiently. The commercially available liposomal formulations like Ambisome^®^, Doxil^®^, Myocet^®,^ etc., cross the BBB by RMT, and the positive charged liposomal formulations reach the brain via AMT. Furthermore, inorganic NPs like gold nanoparticles traverse the BBB by RMT 126.

### Features of Nanomaterials or nanocarriers

The overall goal of nanocarrier-based drug delivery is efficient disease treatment with minimum side effects by taking advantage of the pathophysiology of a diseased tissue microenvironment. For passive diffusion across the BBB, small size molecules with high lipophilicity are ideal. Lipophilicity is thought to be associated with the permeability and solubility of a compound. However, lipophilicity is a double-edged sword. Increased lipophilicity may result in rapid metabolism, low solubility, and poor absorption of the drug. Thus, for the targeted and control release of such molecules, nanotechnology-based DDSs can be a suitable approach 127.

The parameters critical for the application of nanomaterials in practical medicine include (1) size, (2) shape, (3) surface chemistry, (4) flexibility/rigidity, (5) hydrophobicity, (6) architecture, and (7) elemental composition, that should be taken care of to attain a varied array of synthetic nanostructures 128.

NPs are colloidal carriers from natural or synthetic sources and ranges from 1 to 1000 nm in size. NPs include micelles, inelastic spherical shells, nanotubular particles, liposomes, gold NPs, and polymers. The NP can either be encapsulated or conjugated with some of the parent components for the drug delivery. Moreover, determination of polarity, improving lipophilicity, or introducing surface receptors recognizing the specific cell type may aid in enhancing the NP as a drug delivery component. The NP's capsule thickness and the capsule's size (range between 1 and 300 nm) are important factors to improve the therapeutic potential of the formulation. The core to surface ratio depends on the size of NPs. The smaller NPs relate to a smaller core to surface ratio, allowing the immediate release of the drug on breaching the NP's membrane 129. However, larger NPs are not ideal due to limitations such as slow decomposition or drug entrapment into the carrier leading to uneven or inefficient drug delivery. The time of release for a drug is also considered an important feature. The natural method of transcytosis helps improve the specificity of the drug to the target tissue/organ. NPs are versatile to adapt with other cellular components or be fused with them (i.e., antibodies, peptides), which add to the aptness of NPs as drug carriers 17.

### Types of Nanocarriers

**Figure 3:**
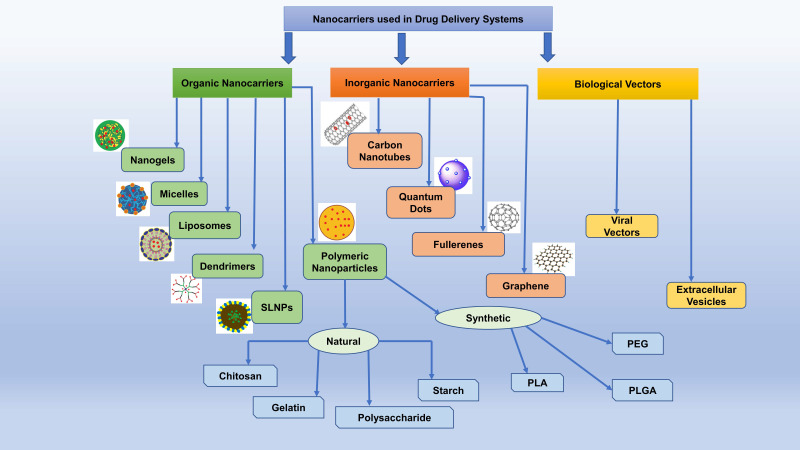
**Classification of Nanocarriers involved in Drug Delivery System**. Based on origin, nanocarriers are classified as organic, inorganic, and biological vectors nanocarriers. SLNPs, solid-lipid nanoparticles; PLA, polylactic acid; PLGA, poly (D.L-lactide-co-glycolide); PEG, polyethylene glycol.

Modern smart nanostructured systems can be broadly divided into organic and inorganic nanocarriers (Figure 3) [Table 4] 130.

#### Organic Nanocarriers

Organic nanocarriers are biocompatible with comparatively high drug holding capacity.

##### Polymeric Nanocarriers

Polymeric nanoparticles (PNPs) involve matrix architectures and are mostly used as nano-capsules or nanospheres in drug delivery. In the current scenario of drug delivery to the brain, PNPs have been extensively used to deliver oligonucleotides, proteins, and small-molecule drugs to treat IS. Biodegradable PNPs possess high biocompatibility, nontoxic by-products within the body, and good sustained-release profiles, making them the potential carriers for drug delivery to the CNS 131. PNP materials are further divided into synthetic biodegradable polymers and natural macromolecular systems. Synthetic biodegradable polymers include PLA, PGA, PLGA, polycaprolactone (PCL), and polyethylene glycol (PEG). The natural macromolecular systems include chitosan, polysaccharide, gelatin, starch, etc. The overall potential of therapeutic delivery with PNPs involves endocytosis or transcytosis via endothelial cells, drug accumulation in the brain capillaries resulting in a high concentration gradient, increased membrane fluidization through lipid solubilization (surfactant effect), TJs opening. To improve the efficiency of PNPs, they can be coated with polysorbates which restricts the efflux phenomenon of the membrane. Furthermore, surface conjugating of PNPs with targeted peptides or cell-penetrating ligands can improve the transcytosis across the BBB 17.

##### Dendrimers

Dendrimers are artificial, highly branched, globular macromolecules, commonly formulated using polyamidoamine (PAA), polypropylenimine, and polyaryl ether (PAE). The structure of dendrimers is a tree-like topological assembly with an initiator core, branched repeat units from the core, and functional groups at terminal ends on the external layer of the repetitive units. Dendrimers are capable of encapsulating hydrophilic and hydrophobic molecules, thus are widely used as nanocarriers to transport various therapeutic and imaging agents. Dendrimers can enter across various cell membranes or biological barriers, including the BBB, through endocytosis. Moreover, to improve brain targeting or transport across the BBB. It has been documented that injected dendrimers are selectively taken up by the ischemic region on their own 130, 132.

##### Nanogels

Nanogels are three-dimensional, water-soluble, and cross-linked polymers with stable encapsulation features, creating a reservoir for the drug and allowing the drug's controlled or sustained release. Although nanogels possess the comparative control release properties, the amorphous structure and high-water content of nanogels contribute to the greater surface area, unique softness, and better drug loading capacity in these formulations in comparison to other DDSs. Until now, the utility of nanogels for stroke therapy has limited preclinical literature. However, nanogels had been extensively studied for thrombolysis in the rat model of IS. In a study, urokinase (UK) was loaded with chitosan nanogel, and ultrasound stimulation was used to dissociate. Likewise, PEGylated UK was used as a nanogel polymer dissociated in low pH in the ischemic microenvironment 133.

##### Micelles

The utility of polymeric micelles as a DDS has been recognized in the last few years. These micelles form spontaneously in amphiphilic copolymer solutions and show shell-core structures. The core consists of hydrophobic block polymers (e.g., L,D-lactine polycaprolactone) and the shell comprises hydrophilic block polymer (usually PEGs). The characteristic particle size of polymeric micelles lies between 10-100 nm 134. The lipophilic drugs are loaded into the core, which improves drug stability and bioavailability. The shell shields the drug against nonspecific interaction with serum proteins and non-target cells and allows the release of loaded drug through diffusion after reaching the target cell. Ding et al. have formed an oral polymeric micelles-based DDS for targeted delivery of borneol. The system consists of a binary micelle combining PEG co-polymers. However, the system has certain limitations, such as poor stability and low drug loading capacity, but it also involves a long-term *in vitro* drug release profile. Interestingly, both mixed micelles and poly(ethylene glycol)-poly(caprolactone) ether (PEG-PCL) micelles have been reported to improve bioavailability in humans and rats 86.

##### Liposomes

Liposomes are spherical vesicles comprising of an aqueous core surrounded with single or multiple amphiphilic lipid bilayers, because of which they can entrap both hydrophilic and hydrophobic compounds, respectively. Thus, the liposomes as nanocarriers can efficiently deliver therapeutic molecules, including drugs, vaccines, enzymes, proteins, and nucleic acids, and imaging agents for diagnostics by transcytosis, endocytosis, and BBB disruption. Moreover, liposomes can cross the BBB via active or passive targeting and deliver the required amount of therapeutic and diagnostic agents to the CNS, thus establishing their potential in neurological applications 131, 135.

##### Solid-lipid nanoparticles (SLNP)

*SLNP* is the next-generation colloidal nanocarriers comprising surfactant-stabilized triglycerides, monoglycerides, complex glyceride mixtures, waxes, hard fats that remain solid at both room as well as body temperatures. The core consists of a hydrophobic solid matrix, where phospholipids are rooted through the hydrophobic tail regions, resulting in enhanced entrapment efficiency for hydrophobic drugs in the core compared to conventional nanocarriers. The size of *SLNP* used for formulation lies between 50 and 1000 nm. *SLNP* possesses the advantages of both liposomes and polymeric nanoparticles while taking care of associated individual disadvantages. Thus, the key characteristics of *SLNP* include; high physical stability, bioavailability, biocompatibility, drug protection, strict control of release, ease of preparation, efficient tolerance, and biodegradability without generating toxic by-products.

Furthermore, the lipophilic nature of *SLNP* helps them access BBB with ease and make them a first-choice nano-vehicles for the delivery of therapeutics to the brain. The key mechanisms by which SLNP uptake by the brain occurs is the paracellular pathway via TJs opening in the brain microvasculature, passive diffusion, active transport, and endocytosis. It is important to note that apolipoprotein E receptors are mostly expressed in the brain. Thus SLNP functionalization with this protein may be a promising strategy in improving drug delivery to the brain 136, 137.

#### Inorganic Nanocarriers

##### Carbon-based Nanomaterials

Carbon-based nanomaterials have attained significant scientific attention in different fields because of their unique structures, exceptional mechanical, thermal, optical, and electrical properties, and high surface area. Their properties involve various biomedical applications like drug hormone and enzyme delivery, gene therapy, tissue engineering, and biosensing. According to previous literature, they cannot cross the BBB via passive diffusion, which indicates the energy-dependent mechanism of these drug delivery systems. Furthermore, carbon-based nanomaterials have ROS scavenging properties. On the other hand, they are also reported to generate ROS due to the presence of heteroatoms. According to a hypothesis, their antioxidant property is based on the formation of radical adducts at sp2 carbon sites and its elimination during subsequent adduct formation by electron transfer or hydrogen donation from functional groups (86,131,132).

##### Fullerenes

A fullerene is a soccer-ball-shaped zero-dimensional carbon-based nanomaterial structure. It is an allotrope of carbon formed as C60 and C70. The shape gives it distinctive surface chemistry providing an extensive yet simple decoration for biomedical applications. Fullerenes are highly respective to radical species due to the abundant presence of conjugated double bonds and low-lying lowest unoccupied molecular orbital (LUMO). This catalytic property also allows a fullerene to react with superoxides without getting consumed. Its ability to absorb electrons and to disperse through 3D- conjugated structure gives it an antioxidant functionality. Their micro molar concentrations help eliminate superoxide anion and H_2_O_2,_ making them act as “free radical sponges” 132, 138.

The activity of SOD may produce H_2_O_2_ in excess amounts. The scavenging activity of fullerene controls this by removing superoxide anion and H_2_O_2._ New evidence shows that alteration of cellular redox state and enzyme activities of fullerene C60 may contribute to cell survival. Polyhydroxylated fullerenes (fullerenol) and carboxy fullerenes are water-soluble that are conjugated with a polar group. These fullerenes can enter the cell membrane localizing themselves to the mitochondria where oxygen free radicals are found. Research has shown that carboxy fullerene prevents disruption and leakage of mitochondrial membrane, an activating step in apoptosis 139. There is a vast application of different derivatives of fullerenes to ischemic tissue for possible effects on reducing ROS and preserving tissue function after ischemia. Thompson et al. report an increase in myocardial infarction size and contraction of coronary artery upon intratracheal or intravenous administration of C60 fullerene. Antioxidant features and biological behavior contributing to cell survival against oxidative stress or enhancing cell death may be determined by the fullerenes' size, structure, and surface 132, 140, 141.

It is the virtue of the antioxidant nature of fullerene that it has been widely used for delivering drugs to the brain. Fullerene effectively crosses the BBB when hybridized with a biologically active moiety, thus making targeted drug delivery readily possible 142. The water-soluble fullerene derivatives are even more potent than fullerene as a drug delivery system in CNS 143. Non-covalent adsorption of several chemotherapeutic agents at the fullerene surface helps enhance the polarity of C60, thus facilitating easy pass from BBB and targeted drug delivery in the brain 144,145.

##### Graphene

Graphene is a 2D single layer of strongly packed carbon atoms. Hydroxyl, epoxyl, and carboxyl groups modify the graphene to provide graphene oxide (GO), giving it various properties against the unmodified graphene. Graphene offers potential for nanomedicine (detection of single nucleotide polymorphisms of DNA, early diagnosis, and treatment) due to its special electron mobility, thermal conductivity, and biocompatibility. In a study, Qui et al. assessed the chemical activity of GO, reduced graphene oxide (rGO), and few-layer graphene (FLG) against ROS. A strong activity against hydroxyl, moderate activity against H_2_O_2_ and LPO along with stable radicals including 2,2-diphenyl-1-picrylhydrazyl (DPPH) and 2,2'-azino-bis-(3-ethylbenzothiazoline-6-sulphonic acid) (ABTS) was shown by all graphene-based materials. Graphene-based materials also prevent phytochemical ROS production by absorbing UV 146. However, in few studies, it was found that GO and rGO were the sources of ROS generation. Also, the procedures applied for the GO reduction effect and controlled the ROS level produced by rGO. Hence, electron density, chemical features, and sp2-hybridized carbon content determine graphene-based materials' antioxidants. Using stem cell-based tissue engineering, graphene can be applied to any tissue injury. For example, the survival of mesenchymal stem cells (MSCs) when implanted to treat myocardial infraction reduces due to ROS generated in the ischemic myocardium followed by reperfusion. According to the study, the implanted MSCs from ROS mediated death after MI by GO flakes through their antioxidant mechanism. Another form of graphene called Graphene foam has a 3D architecture 147. Lately, it has attracted much attention as a neural interface material due to its topographical properties that can trigger anti-inflammatory responses by restricting the microglia morphological transformations on its surface. In addition, a close link is found between antioxidant and free radical scavenging effects of graphene foam and its sp2-carbon network. In Wnt signaling pathway-dependent mechanism, it is seen that the synergistic antioxidant effects of vitamin C and graphene foam saved the H_2_O_2_ impaired cell viability and differentiation of human bone-marrow-derived MSCs under oxidative stress 148, 149.

In addition to its antioxidant properties, graphene in the form of graphene oxide has been utilized by researchers to play the role of nanocarrier in drug delivery to the brain. Several therapeutics are linked to graphene oxide for easier transport to the targeted brain site 150, 151. Moreover, these novel nanocarriers are highly specific in their targeted drug delivery, making them more applicable in medicine. In their study, Joo et al (2016) successfully synthesized and characterized a graphene oxide encapsulated nanocarrier system designed for the slow and delayed delivery of siRNA to injured brain regions in mice 152. In a similar study, a magnetic nano-graphene oxide-based nanocarrier system was designed to achieve high drug accumulation and enhanced therapeutic efficiency in the brain 153.

##### Carbon Nanotubes

Carbon nanotubes (CNTs) are cylindrical-shaped carbon-based nanostructures. They can be either single or multi-layered, called single-walled and multi-walled CNTs. These are valuable tools in medicine as they have unique chemical, mechanical and electrical properties. Evaluation of pure and modified forms of CNTs has revealed that the formation of nanotube-neural hybrid networks promoting neuronal activity, network communication, and synaptic formation 154. A new prospect for applying CNTs is in the design and fabrication of nervous tissue by cellular stimulation due to maintenance of the interaction between CNTs and stem cells. Kafa et al. showed in an animal model using a scanning electron microscope that a high amount of CNTs accumulates in the brain tissue and uptake by astrocytes by studying the permeability of single-walled CNTs conjugated with an amino group 155. It was also observed that with the increase in temperature, there is a decrease in the permeability of these nanostructures, indicating the energy-dependent mechanism of these DDSs 85.

##### Quantum Dots

Quantum dots are zero-dimensional nanomaterials, which have fascinated significant scientific interest due to their extraordinary optical and electrical features. In medicine and biology, their application has arisen as nano-scaled systems for targeted drug delivery, imaging, diagnosis, and transplanted labeled cells for tracking. However, a subsequent surface functionalization is required by quantum dots through which brain targeting and BBB crossing could be possible. Thus, the carrier-mediated transport mechanisms are involved in reaching the brain parenchyma 156, 157.

#### Biological Vectors

##### Viral Vectors

The ability of viruses to enter and insert genetic material into the host cell has rendered its use as a viral vector. The use of viral vectors in the CNS is through gene therapy, involving the delivery of a normal copy of a defective gene and subsequently reducing the harmful functions. Retrovirus vectors, adenovirus vectors, lentivirus vectors, herpes simplex virus type 1, and adeno-associated virus vectors (possesses transgene capacity and expression properties) are extensively used and studied in clinical trials for gene therapy 158. Receptor-mediated pathways across the endothelial cells via transcytosis and transient disruption of the BBB are the main strategies for transporting the viral vectors in the brain. Intravenous administration of a highly concentrated mannitol solution resulting in the osmotic shrinkage of cells is one of the methods of disruption. Several studies have shown the utility of herpes simplex viral vectors in fighting stroke through repairing or replacing genes that cause neuronal damage. Using the adenovirus-mediated vectors produced similar results due to ethical risks and high risks of failure 131, 159.

##### Extracellular Vesicles

Extracellular vesicles are a class of heterogenous cell-derived membrane structures. These are called exosomes if they originate from the endosomal system and micro vesicles or from shedding the plasma membrane. These cysts are universally found in biological systems and constitute many roles in intercellular communication throughout the body, leading to protein, lipid, and genetic material exchange. Adsorptive-mediated or receptor-mediated transcytosis are the main pathways for crossing the BBB by extracellular vesicles 160. The exact mechanism of alleviating in physiology and pathological conditions are yet to be fully understood. One of the applications as nanocarriers for brain disorder is through autologous exosomes containing glyceraldehyde-3-phosphate dehydrogenase can deliver small-interfering ribonucleic acid (RNA) to neurons, microglia, and oligodendrocytes. For inhibiting brain inflammation and autoimmune responses, the use of intranasal administration of curcumin-containing exosomes has been reported 161, 162.

### Toxicity Risks of Nanocarriers

Despite being a strong tool to enter the BBB, nanocarriers have several issues that need to be sorted. First, the precise spread in the brain after its absorption is not clear in much research, making it potentially hazardous for the brain. Second, most of the nanocarriers are inorganic materials like silica nanoparticles, gold nanoparticles, cerium oxide nanoparticles, molybdenum nanoparticles, and iron nanoparticles can accumulate in the brain due to its difficulty in metabolization, causing neurodegeneration through malfunctioning of mitochondria, redox alterations and apoptosis, autophagy and reduced lysosomal activity, cytoskeletal impairment, and vesicle trafficking disturbance, neuroinflammation, and microglia activation. In chicken embryos, neuronal loss is caused by magnetic iron oxide nanoparticles, while cerium oxide nanoparticles inhibit the differentiation of neural stem cells.

Research has also reported neurotoxicity caused by biodegradable nanoparticles. For instance, a decrease in body weight was found when polysorbate 80-modified chitosan nanoparticles were injected in a dose-dependent manner for seven days, followed by apoptosis, necrosis of neurons, and a minor inflammatory response in the frontal cortex. Also, 200 nm chitosan nanoparticles cause malformations along with a bent spine, pericardial edema, and the formation of an opaque yolk in zebrafish embryos.

The third issue about nanocarriers is the administration mode. As the majority of the nano-formulations are injections, it would be much easier for it to be in oral dosage forms for patients with neurodegenerative diseases.

Therefore, additional research is needed to study acute toxicity, probable long-term neurotoxicity, and utility 163.

## Routes of administration of NPs for targeting brain

The NP's surface charge, size, and lipophilicity are crucial factors in determining the biodistribution of NPs encapsulating drugs targeted for different body organs 164, 165. As described in previous sections, NPs are categorized into several broad classes based on their structure and functionality. Likewise, these factors also decide the best route of administration for a particular NP type. Extensive studies have been performed recently to determine the most effective route of administration for NP depending on the kind of therapeutic targets. For instance, nanotherapeutics used as anti-cancer agents are best administered through parenteral routes 166, 167. However, the delivery of drugs to the target brain has been a great challenge for researchers due to the biological constraints posed by BBB. Recent advances in nanotechnology have overcome these challenges with great efforts to provide comparatively easy ways to deliver drugs directly to the brain via different routes by changing NP structures. Recent research has provided novel insights regarding different routes of administration suitable for brain-targeted delivery of drugs as described below [Table 5].

### Oral Administration

It is the most acceptable route of administration for drugs due to the ease of application. However, the stability of drugs in the GI tract has been a difficult task for researchers 168. Innovative nanocarriers have been designed recently based on important parameters such as stability in gastrointestinal fluid, large surface area, and minimal irregular adsorption for drug delivery 169. Several studies have highlighted the use of SLNPs to treat cerebral ischemia via oral administration of the ischemic drug.

Delivery of SLNP formulation of methylthioadenosine via oral treatment has proved to be highly effective for treating multiple sclerosis-like conditions in mice models 170. Kakkar et al., in their study, have demonstrated the effective delivery of curcumin encapsulated in SLNP coated with Tween 80 to the brain in rats via oral route for the treatment of reperfusion injury 102. Another study exhibited the successful delivery of SLN encapsulating Resveratrol by oral administration for alleviating the mitochondrial oxidative stress level post-ischemic stroke 170. Additionally, nano encapsulated Quercetin has been proven effective in combating reperfusion injuries in young rats when delivered through the oral route 103. Likewise, oral treatment with NPs containing Puerarin has attenuated the oxidative, inflammatory, and apoptotic effects post-ischemic stroke in the MCAO rat model 171.

### Parenteral Administration

Delivery of drugs through parenteral administration like intravenous and intraperitoneal injections has great merits, such as masking from first-pass metabolism and the highest bioavailability of drugs directly delivered in blood 166. Recently, nanocarriers like SLNPs and gold NPs have been explored for parenteral administration. Zhang et al. (2010) demonstrated the lowest toxicity of gold NPs using intravenous injection (in the tail of mice) 172, 173, 175. Extensive studies have been performed to explore the potential of SLNPs for administration through this route for the treatment of CNS disorders. Hydrophilic coating of SLNPs improved the drug transport through BBB to treat several neurological disorders 174, 176. For instance, the bioavailability of Doxorubicin is increased post-administration with stealth SLNP coated with PEG 2000 due to easy permeability from BBB 177.

### Intranasal Administration

**Figure 4:**
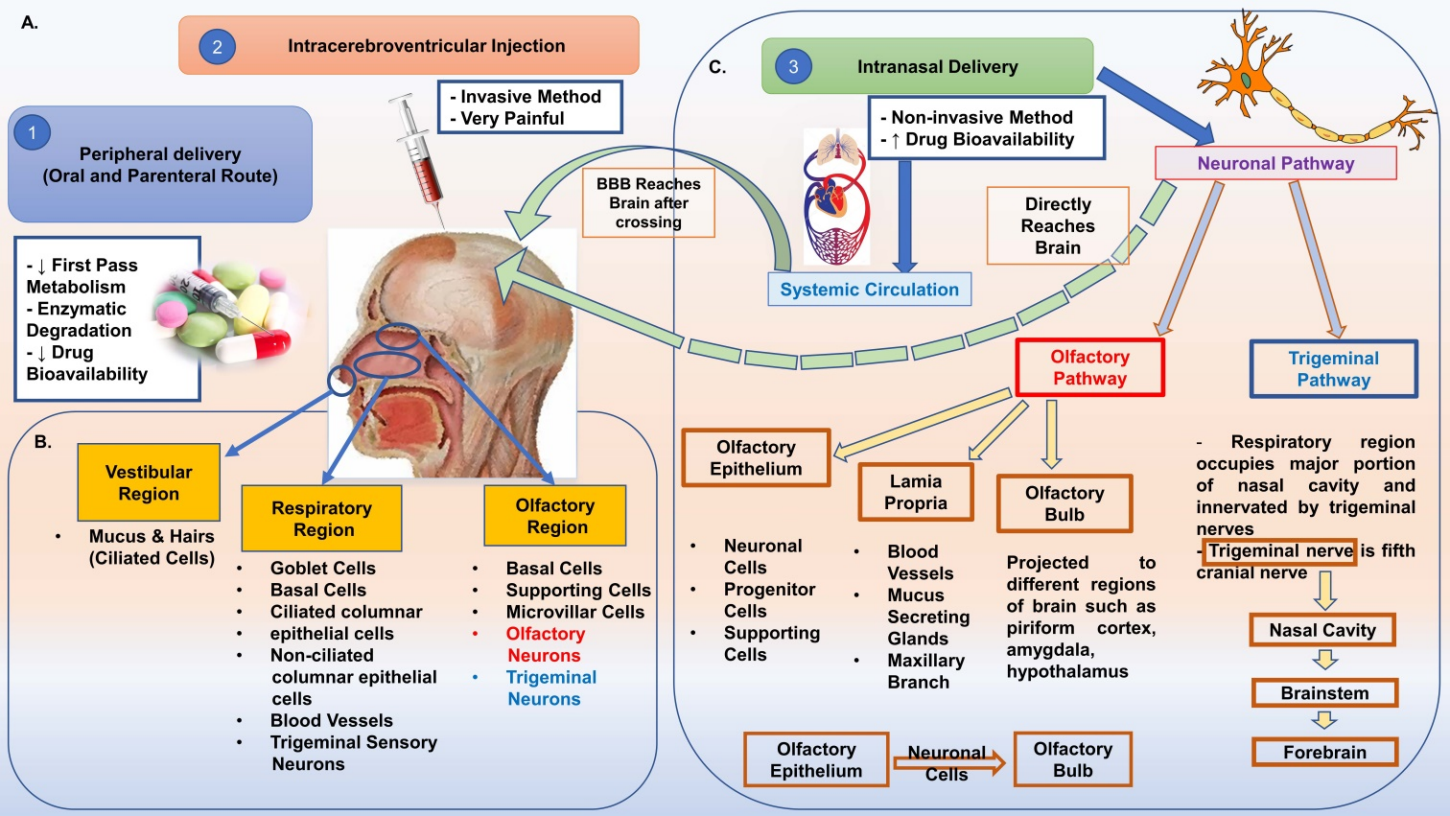
**A. Routes of Administration**. There are three main routes of NPs drug delivery to the brain (1) Oral and parenteral, (2) Intracerebroventricular injection, and (3) Intranasal. **B. Role of a different region of Nasal Cavity in IN DDS.** Intranasal DDS comprises two pathways that involve three regions of the nasal cavity, i.e., Vestibular Region, Respiratory Region, and Olfactory region. The olfactory (major) and trigeminal (minor) play a significant role in drug delivery to the brain via the nasal route. **C. Intranasal Pathway for Drug Delivery to Brain.** Intranasal drug delivery bypasses the blood-brain barrier (BBB) due to the presence of olfactory nerves (Olfactory Pathway) and trigeminal nerves (Trigeminal Pathway). These nerves provide direct access to broad regions of the brain without any hindrance being posed by BBB. The intranasal pathway also involves a secondary route via systemic circulation, which involves the crossing of BBB. (**Major pathway and neurons**- Red color; **Minor pathway and neurons**- Blue color)

The majority of drugs used for the treatment of CNS disorders like Alzheimer's disease (AD), Parkinson's disease (PD), Ischemic Stroke, etc., use peripheral routes of administration (oral and parenteral). However, these routes have a major drawback of limited accessibility of drugs to the brain due to the short half-life of the administered drugs and fight against BBB permeability 166. IN route overcome these limitations easily as this route bypasses the BBB, and the drug is delivered directly into the deep regions of the brain from the nose. It has also been claimed that IN route overcomes the complexities of systemic drug delivery and enhances the overall administration process, thus acting as the best alternative non-invasive method of drug administration 115. The olfactory-neuro epithelium is the faster and easier way to bypass the BBB as it is the only region of the brain devoid of BBB protection 169. The drug delivered to target brain via IN route follows three pathways: 1) Olfactory Pathway involving drug delivery through olfactory neurons bypassing BBB; 2) Trigeminal Pathway involving delivery of drug through trigeminal nerves to the olfactory bulb followed by the brain stem and; 3) Vascular Pathway involving drug delivery through lungs by crossing the BBB (Figure 4) 169. 1 However, IN route has potential limitations such as reduced residence time, nasal irritation, difficulty in transporting high molecular weight drugs, and access to limited brain regions 175. NP-based drug delivery has proved to be highly effective and suitable for IN administration of drugs as they increase the drug loading capacity and bioavailability. Several studies have been performed recently exploring the therapeutic efficacy of anti-inflammatory and anti-oxidative drugs encapsulated in NPs to treat neurological disorders like ischemic stroke and AD. Chitosan-based NPs, SLNPs, PEG-PLGA coated NPs; Polymeric NPs are widely used for direct nose to brain drug delivery 165, 169. Ahmad et al. 2016 demonstrated the preparation of Rutin encapsulated Chitosan-based NPs in their study. These NPs effectively increased the brain targeting efficiency, drug bioavailability, and locomotor activity of rats post-ischemic stroke when administered through IN route in the MCAO rat model 176. A similar study established the efficacy of polymeric NPs encapsulating curcumin, demethoxycurcumin, and bisdemethoxycurcumin in treating cerebral ischemia. These NPs could also prevent the MCAO rats from reperfusion injury, establishing their strong role as neuroprotective agents in ischemic stroke 177. Recently, SLNP has been established as a great alternative for easy transmucosal transport of drugs. Coating of SLNP surface with hydrophilic substances and PEG often enhances their ability to interact with the mucosal epithelium and ease their way for transmucosal transport as drug carriers 173.

Despite certain limitations, the IN route is considered the most suitable and effective route of drug administration for the nose to brain targeting. Further, the advent of nanotechnology has reduced its limitations, making it easier to deliver nano-based neurotherapeutics to the brain regions.

## Devices for targeting brain via nasal drug delivery

**Figure 5:**
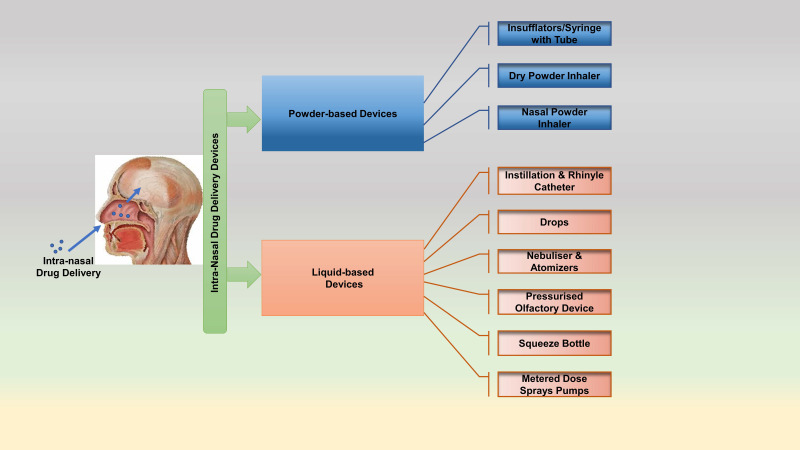
Diagrammatic representation of intranasal drug delivery devices.

Novel strategies have been devised nowadays for direct drug transport from nose to brain. The plan is to deposit the drug on the olfactory epithelium or nose region rich in trigeminal nerves for easy transport through the olfactory or trigeminal pathway to the brain. To execute the same, several efficient novel drug delivery devices have been invented by researchers lately. Atomizers, nebulizers, pressurized meter dose inhalers, pressurized olfactory delivery devices, and powdered devices have gained clinical access (Figure 5). These devices are further categorized into different classes, namely, powder, liquid, and semi-solid formulations, as described below.

### Powder devices

Powder particles interact with nasal mucosa for a longer time duration as they are not easily soluble in the nasal mucosa. This acts as great suitability of powder formulations for intranasal drug delivery. Macromolecular drugs consisting of a large chain of peptides and non-peptides have been successfully designed as powder formulations by researchers. Moreover, mucoadhesive powdered polymers contained in metered-dose insufflators are extensively used to deliver polymers in the nasal cavity. These further results in the formation of thick gel of polymers on interaction with mucosa, thus reducing the clearance from the nose due to mucociliary action 115.

#### Insufflators/syringe with tube

In the absence of pathological conditions, this device is often suitable for direct drug delivery to the olfactory region. It consists of a drug contained in a tube/straw attached with a syringe for easy delivery. Before application, local anesthesia is required to facilitate drug delivery to the olfactory region 115.

### Dry powder inhaler

Dry powder inhalers (DPI) consist of drug particulates either suspended in a propellant or dissolution of the drug occurs on contact with nasal mucosa and are suitable for single use. To avoid irritation and cough, small doses in milligrams are delivered in such inhalers. DPIs are further classified into mono and multi-dose inhalers. Accurate drug dosing is achieved with the help of multi-dose inhalers. On the other hand, mono-dose inhalers are single-unit syringes 179.

#### Nasal powder sprayers

This device is often used for systemic delivery, suitable for low and high molecular weight drugs. Nasal powder sprayers do not require absorption enhancers because the carriers facilitate prolonged drug contact with mucosa and thus directly increase drug absorption. 179

### Liquid-based devices

Another drug delivery device class is liquid-based formulations, consisting of a suspension, liquid solution, or emulsion of drug formulations. The formed drug solutions are then delivered in nasal sprays, nasal drops, and metered nebulizers. These devices are widely used in pathological conditions due to their humectant nature, thus preventing mucosal dryness. However, liquid formulations have certain limitations, such as instability due to microbial growth and preservatives, which further cause irritation and allergy. Additionally, drugs are less stable in liquid formulations as they tend to get easily washed from nasal mucosa due to mucociliary action.

#### Instillation and rhinyle catheter

Catheters are the most commonly used technique for delivering liquid formulations of the drug to specific nasal cavity regions by inserting it in the nostril and delivering liquid in the defined region. However, the risk of mechanical injuries due to sensitive human mucosa and inconsistency of doses are major limiting factors for the clinical use of this technique 180. Thus, this is mostly used only for drug delivery to rodents. Nevertheless, rhinyl catheters are used in some countries to treat nocturnal enuresis, diabetes insipidus, and Von Willebrand disease.

#### Drops

Drops are yet another easy way for intranasal drug delivery of liquid-based formulations. Drops are administered in two ways: 1) Inserting a liquid-filled glass dropper in the nostril and pressing its rubber top to easily release the drug, a method feasible for single-dose administration. 2) Use of metered spray pumps for precise multi-dose administration. However, expensive spray pumps are being replaced with economical disposable pipettes for clinical use.

Although drops are economical and easy to manufacture, they contain a high risk of chemical instability and microbial contamination 119, 181.

#### Squeeze bottle

Drug delivery to local nasal regions could be done easily using squeeze bottles. They comprise plastic bottles partly filled with air, connected to a nozzle and an outlet. Specific drug volume is emitted from the nozzle into the nasal cavity by pressing the bottle. Release of pressure again fills the air back in the bottle. However, squeeze bottles are ineffective for brain-targeted delivery due to their incapacity to reach deep nasal cavity regions. Also, the device is highly susceptible to contamination as a result of nasal secretions 182.

#### Metered-dose spray pumps

Metered spray pumps are effectively used for the local and systemic delivery of antihistamines, nasal decongestants, and corticosteroids. They are efficiently used for consistent and precise dose administration. They are made up of three parts: a container, an actuator, and a valved pump. These pumps generate a fine mist, and the drug is instilled into the nasal cavity. Deposition of the drug is dependent on the design of the spray device, droplet size, viscosity, etc. Unfortunately, metered spray pumps can deliver only a small volume of drug, with only 2.5% of administered volume reaching the olfactory region. Thus, these are inefficient for brain-targeted delivery 119.

#### Pressurized olfactory device (POD)

Consistent delivery of therapeutics to the olfactory region has been challenging for researchers due to its location in the upper nose region with turbinate restriction. However, POD successfully deposits most drug molecules at the olfactory epithelium, facilitating direct transport of drugs to the brain. Moreover, this enhances the drug distribution to the brain along with systemic absorption of the drug. Another merit of POD is that it could be efficiently used to transport both powder and liquid formulations through nasal epithelium 119, 183.

#### Nebulizers and atomizers

Compared to traditional nasal sprays, nebulizers are highly effective for nose-to-brain drug delivery. They enable the deposition of drugs molecules to the intricate nasal regions housing neural connections between the brain and nose. Nebulizers work by breaking liquid solutions into aerosol droplets of small size through ultrasonic power or compressed gas. IN atomizers are used as an alternative to intravenous delivery. Atomizers could efficiently deliver a wide variety of drug formulations with varying surface tensions and viscosity. They could easily target the upper parts of the nose for the nose-to-brain targeted drug delivery. Additionally, jet nebulizers and vibrating mesh are gaining attention nowadays to examine the olfactory deposition of aerosol medicines. 184, 185, 186

## Nano-theragnostic applications for stroke diagnosis and management

### Diagnostic application

Presently, stroke diagnosis and management are made clinically based on the patient's medical history and physical examinations. This is followed by neuroimaging examinations such as CT scans for assessment of suspected stroke patients. However, a non-contrast CT scan is an insufficiently sensitive method for detecting ischemic stroke as it poses several limitations. As discussed, a density of a clot is similar to the density of surrounding blood. This makes it extremely difficult to differentiate an ischemic stroke from neurological symptoms, which are just mimicking the stroke symptoms, such as in case of migraine, brain neoplasms, hypoglycemia, etc. Thus, a need arises for a potential method to overcome this limitation. Kim et al. conducted an experiment involving the use of gold NPs in brain imaging, providing strong evidence that their study overcame the limitations of normal imaging techniques by reducing the side effects and drastically enhancing thrombi detection 187, 188, 189.

### Profiling of Molecular Biomarkers

ROS secretion followed by the release of pro-inflammatory cytokines due to glutamate excitotoxicity in ischemic stroke results in apoptosis or necrotic cell death. Hence, early detection of levels of ROS in the brain could be a strong indicative factor paving the way for immediate therapeutic interventions and stroke management 189. NPs have proved to be effective for easy detection of ROS in several studies conducted by researchers. For example, Hyun et al. successfully demonstrated the use of fluorescein-labeled hyaluronic acids (HA) on the surface of gold NPs to detect ROS in the MCAO rat model of IS. This study provided strong proof that fluorescein-labeled gold NPs are an excellent tool for diagnosing the ischemic disorder. Furthermore, they are highly sensitive to trace levels of ROS up to 41h post-ischemia in rats 190.

Apart from ROS, thrombi are another important pathophysiological feature of IS. Identification of blood biomarkers such as D-dimer and prothrombin fragment 1.2 are recent stroke management approaches for detecting thrombin activity 189, 191. However, this technique lacked the specificity required for biomarker detection. A study conducted by Lin et al. overrides this limitation by engineering NPs highly sensitive and specific to thrombi detection and releasing reporters into the urine of thromboplastin-induced pulmonary embolism mouse model 192. This approach successfully established novel insights on the use of NPs in the diagnostic application of IS and its management.

### Targeted Imaging

Accurate diagnosis of neurological disorders such as stroke demands high resolution and sensitive visualization of the diseased site in the brain, along with continuous monitoring for its effective management. This requirement is fulfilled by the use of several imaging techniques, such as Computed tomography (CT), magnetic resonance imaging (MRI), Single-photon emission computed tomography (SPECT), Positron emission spectroscopy (PET), ultrasound, and optical imaging 193, 194. However, similar to other techniques, these advanced techniques also suffer from certain limitations like bad contrast due to interference from biological entities or fuzzy/ambiguous images, making it difficult for clinicians to differentiate between normal and pathological tissues. However, the advancement of nanotechnology has provided novel insights on the production of better imaging via contrast enhancers. Additionally, nanocarriers are explored for the encapsulation of these contrast agents for better accuracy in diagnosing pathological conditions 195. Moreover, the improved lifespan of contrast agents, evasion of physiological barriers in diseased tissue, and target-specific accumulation have also been achieved with the help of nanotechnology.

#### Magnetic resonance imaging (MRI)

A non-invasive technique widely used for the imaging of the brain without using ionizing radiations. MRI generates intensity maps of the tissues based on the relaxation intensities of the bound form of water and free form of water associated with membrane structures and extra/intra-cellular matrix. However, since the intensity difference is not great, images generated are often fuzzy. This drawback could be easily overcome by the use of NPs of paramagnetic molecules as contrast enhancers. These NPs, in contact with the bound form of water molecules, change their relaxation time, thus altering the intensity differences and producing high contrast, quality resolution, and high sensitivity images 196.

#### Computed tomography (CT)

A usual CT scan image amalgamates several X-ray images of the brain taken at different angles. As compared to MRI, it is less sensitive for differentiating normal and ischemic brain tissue. On the other hand, CT angiography is a better technique for imaging occlusions among large vessels.

Routine use of NP-based contrast enhancers is being done to improve image clarity and resolution. For diagnosing cerebral ischemia, wide use of xenon gas and iodine/iodinated compounds as contrast enhancers have also been done in CT scans. However, the anesthetic effects of Xenon and the low k absorption edge value of iodine limiting the image resolution created the need for NP-based contrast enhancers 196, 197. Facilitation of site-specific visualization of the diseased brain region and ensured crossing of physiological barriers has been achieved by encapsulating the CT contrast enhancers in nanocarriers like PEGylated BaHoF5 nanoprobes. These probes have been employed for CT angiography and CT perfusion imaging, providing high contrast images of the specific brain region even at lower doses than iodinated contrast agents 198. Similarly, gold NPs are widely accepted as CT contrast agents because of their biocompatibility and high k absorption edge values. Gold NPs incorporated into polymeric NP systems further provide great avenues for therapeutic, imaging, and targeting moieties.

#### Positron emission tomography (PET)

PET is used to map the metabolic and physiological markers in the targeted region. The mapping is done by employing a radioactive tracer to examine the brain's functioning. Gold NPs are commonly employed in PET, followed by Copper Sulfide NPs. The other NPs used are farnesylthiosalicylate- based copolymer, carboxymethylcellulose- based NPs, ^18^F- Macroflor modified polyglucose NPs and cerium oxide NPs. PET is often used to identify regions where the tissue functions are compromised over the preserved morphological aspects 199. The clinical utility of PET is limited, though it is widely used for research purposed on several brain disorders. This technique is used in combination with MRI and CT scans for enhanced imaging purposes.

#### Ultrasonography

Reflection of ultrasound waves (echogenicity) by body tissues forms the basis of ultrasonography. It is widely used to detect the blockages in blood vessels since they possess poor echogenicity and poorly reflect the ultrasound waves. Thus, contrast enhancers comprising of gas-filled microbubbles are used as they have strong echogenicity. Few of the examples of NPs used in ultrasonography are silica NPs, iron-silica NPs (Fe-SiO_2_), CaCO_3_ hybrid NPs, hollow mesoporous Prussian blue NPs filled with perfluoropentane, supermagnetic iron oxide NPs, hyaluronic acid NPs. Furthermore, ultrasonography can easily restrict specific organ features along with the measurement of blood flow characteristics. This provides great merit of this technique when compared to MRI and CT 196, 200.

#### Fluorescence Imaging

Recently, non-invasive optical imaging has become a fascinating approach to studying the various classes of structures involved in autophagy at macroscopic and microscopic dynamic levels. Fluorescence imaging is widely preferred by researchers in a biomedical imaging application, as it provides good results in less time and can be interpreted easily. In biomedical studies, the molecules are localized and highlighted with the help of fluorescent NPs. In fluorescence imaging, the wavelengths for excitation and absorption should be in the near-infrared (NIR) region to better detect signals. The optimal range for excitation and emission are 700-750 nm and 750-800 nm, respectively. Fluorescence imaging is widely used in biomarker analysis, diagnostics, and immunoassays but has drawbacks like photobleaching and fluorescence quenching 211.

## Future Directions

IS is considered a medical emergency in which interruption of blood supply leads to damage to the brain. Despite being a major concern for society, there is no particular medication for the treatment of IS except t-PA. Therefore, there is a need to develop medications that fulfill the needs and cater to proper treatment with minimum or negligible side effects and high potency. However, there are numerous drugs with the possible capability to restrict the pathological events of IS that are still under inquisition to find an appropriate delivery system or strategy for effective transport to the brain. The maximum of the neuroprotective agents are macro-biomolecules which usually fail to cross the BBB. Lack of strategies for effective drug delivery is a major challenge for advancing efficient treatment for stroke. Nanomedicine is an attractive approach for targeting multiple cascades of IS pathology. NPs are a good alternative in terms of improved stability of drugs, blood circulation, penetration of BBB, and targeting of infarct region. The IN route of drug delivery offers a direct and effortless approach for stroke medication. Over the years, scientific investigations showed fruitful results that confined within the laboratories. For clinically successful development of IN nanoformulations, in-depth understanding of the drug distribution pattern, focusing drug exchange with parenchyma and cerebral spinal fluid is essential.

Polymer biodegradation rate, retention time at the infarct site, and drug leakage are crucial for designing efficient DDS. Also, a uniform approach for studying the pharmacokinetic response of human studies is required. An in-depth understanding of the mechanism involved in the drug delivery from the nasal route to the brain is also required to develop an appropriate device that helps in drug targeting to the specific region. Further detailed studies should be focused on the fate of NPs after IN drug delivery and the cerebral metabolism mechanisms that will successfully help translate DDSs and drug-embedded NPs from bench to bedside and eventually improve the treatment and management of cerebral ischemia. In addition, further studies are required to compare the nature and efficacy of various nanocarriers used in DDS in treatments related to IS to move a step ahead in finding the best method for targeting IS.

## Concluding Remarks

Aspects like a therapeutic window, physiological barriers, and impairment of brain function make ischemic stroke a major challenge for clinicians. The practice of nanomedicine for delivering neurotherapeutic drugs for the lyses of clot and alleviate reperfusion mediated neuronal injury, and restricting oxidative stress is attaining momentum as a possible option to conventional therapy. The intranasal drug delivery system is showing promising results compared to oral and parenteral routes of drug administration. Research is undergoing to develop novel intranasal drug delivery devices with low side effects and better delivery systems. In addition, nanoparticle-based techniques for diagnosing and visualizing infarct areas with high resolution are gaining attention. Developing novelties are focused on designing multi-functional carrier systems and co-encapsulated drugs that target thrombolysis and neuroprotection. However, it is challenging to overcome the critical issues involved in selecting carriers, integrating various functionalities, and identifying the suitable route of administration; nanomedicine is the hope for a new era in the management of ischemic stroke.

## Figures and Tables

**Table 1:** Strategies available for treatment of ischemic stroke.

S. No.	Strategy for IS Treatment	Characteristic Features	Shortcomings	References
1.	Thrombolysis	Intravenous administration of recombinant tPA (rt-PA or Alteplase)	tPA is found to have neurotoxic properties in several studies	49, 74
2.	Mechanical Thrombectomy	Elimination of clot in addition to thrombolysis	Complications related to vascular access, radiological injury, device related vascular injury	56
3.	Anti-Coagulant and Anti-Platelet Therapy	Clot dissolution and anti-platelet drug administration at the same time	Symptomatic intracranial haemorrhage	62, 63
4.	Neuroprotection	Arrests pathological events of IS; Promotes mitochondrial function and anti-inflammatory responses	Lack of efficacy; Need to be used in amalgamation with other available strategies for treatment of IS; Failure in clearing clinical trials	13, 68, 77

**Table 2:** Comparative analysis of anti-stroke drugs and their nano formulations.

S. No.	Drug	Nanocarrier	Surfactants used for nanocarrier	Animal Model	Route	Dose		Molecular Target(s)	Significant Findings		Ref*.*
						Free Drug	Drug-NP		Free Drug	Drug-NP	
1.	Curcumin	SLNP	Tween 80 and Lecithin	Male Wistar Rats; BCCAO (*n=6*)	Oral	25 & 50 mg/kg b.wt.	25 & 50 mg/kg b.wt.	Xanthine/Xanthine Oxidase System; Other ROS systems	No effect on body weight & temperature in I/R group No effect on locomotor activity in I/R group Increased memory consolidation in MWM No significant effects on levels of SOD, catalase, GSH, mitochondrial complex enzymes, LPO, nitrite and AChE levels Poor bioavailability due to first pass metabolism	Increased body weight and restored normal temperature in I/R group Significantly restored locomotor activity in I/R group Better memory consolidation in MWM as compared to free curcumin treated group Significant increase in levels of SOD, catalase, GSH & mitochondrial complex enzymes and decrease in LPO, nitrite and AChE levels 16.4 times improvement in brain bioavailability as compared to free curcumin	102
2.	Curcumin/Demethoxycurcumin/Bisdemethoxycurcumin	Polymeric N-isopropyl acryl amide (PNIPAM)	-	Wistar Rats; MCAO (*n=6*)	IN	100 µg/kg b.wt.	100 µg/kg b.wt.	Antioxidant System	Improved locomotor activity Improved grip strength Decreased TBARS level Protection to antioxidant enzymes such as SOD, catalase, GPx and GR	Significant changes were observed for all behavioral and oxidative stress parameters Antioxidant potential of nano-formulations is: Curcumin>Demethoxycurcumin>>Bisdemethoxycurcumin Significant prevention from I/R injury as compared to other groups	177

3.	Resveratrol	SLNP	Lecithin	Male Sprague Dawley Rats; BCCAO (*n=5*)	Oral	10 mg/kg b.wt.	10 mg/kg b.wt.	Nrf2/HO1 pathway; Mitochondrial Oxidative Stress	Bioavailability in brain: 7.01 ± 0.53 μg/g	Bioavailability in brain is higher than free RSV: 31.37 ± 0.32 μg/g Significant reduction in escape latency and path length and 2.5-fold increase in path efficiency as compared to BCCAO rats in MWM experiment Significant reduction in mitochondrial ROS production, LPO and protein carbonyls in BCCAO rats Significant reduction in HIF-1α levels Significant increase in Nrf2 and HO-1 levels	173
4.	Panax Notoginsenoside	Hybrid Liposomal Vesicles	Polyvinylalcohol	Male Sprague Dawley Rats; BCCAO (*n=10*)	Oral	30 mg/kg b.wt.	30 mg/kg b.wt.	Antioxidant System	Attenuation of brain infarction induced by I/R injury No significant restoration of antioxidative enzymes levels	Significant attenuation of I/R induced brain infarction as compared to free drug solution Significant restoration of antioxidant enzymes levels near to normal levels such as LDH, MDA, H_2_O_2_ and SOD.	201
5.	Quercetin	Polylactide-co-glycolide (PLGA)	-	Male Sprague Dawley Rats (Young and Aged); BCCAO (*n=6*)	Oral	2.7 mg/kg b.wt.	2.7 mg/kg b.wt.	Mitochondrial Antioxidative System	No significant downregulation of iNOS and caspase-3 activities No significant improvement in neuronal cell count in hippocampal CA1 and CA3 regions in both young and aged rats	Significant downregulation of iNOS and caspase-3 activities Improved neuronal count in hippocampal CA1 and CA3 region in both young and aged rats Complete protection to mitochondrial membrane in young and aged rats' brain regions	103
6.	Puerarin	Hydroxypropyl beta cyclodextrin	-	Male Wistar Rats; MCAO (*n=8*)	Oral	05 mg/kg b.wt.	05 mg/kg b.wt.	BBB permeability	Poor bioavailability and BBB infiltration Reduction in mean infarct volume in MCAO rats as compared to control group Improvement in cortical EEG power, peak, area and frequency as compared to control group	Enhanced drug bioavailability and BBB penetration Significant reduction in mean infarct volume in MCAO rats as compared with control and free drug groups Significant infiltration of inflammatory cells and alleviation of neuronal pyknosis and karyolysis Significant mitigation of cell apoptosis Significant improvement in cortical EEG power, peak, area and frequency in comparison to control and free drug group	202
7.	Rutin	Chitosan	TPP	Wistar Rats; MCAO (*n=6*)	IN	10 mg/kg b.wt.	10 mg/kg b.wt.	BBB permeability	Nose to brain direct transport percentage: 29.48 ± 1.05% Poor brain bioavailability Significant improvement in locomotor activity and grip strength of MCAO rats as compared to control group only	Nose to brain direct transport percentage: 93.00 ± 5.69% Significant improvement in brain bioavailability, locomotor activity and grip strength in MCAO group as compared to free rutin application intranasally	176
8.	C3 siRNA	Cationic lipid assisted PEG-PLA	-	Male C57BL/6 J mice; MCAO (*n=3*)	IV	01 mg/kg b.wt.	01 mg/kg b.wt.	Microglia	Reduced localization of C3 siRNA in ischemic region No significant decrease in the levels of inflammatory cytokines such as TNF-α No major effect on pro-apoptotic factor like caspase-3 No significant reduction in infarct size post I/R insult	Inhibited the *in vitro* increase of microglial C3 expression induced by hypoxia/re-oxygenation BBB penetration and delivery of C3 siRNA in ischemic region Significant reduction in microglia induced neuronal damage and TNF-α levels post I/R injury Prevented apoptosis and reduced ischemic penumbra Substantial improvement in functional recovery post ischemia	203
9.	TNF-α	Poly(ethylene glycol)-b-(poly(ethylenediamine l-glutamate)-g-poly(l-lysine) PEG-b-(PELG-g-PLL)	-	Male Sprague Dawley Rats; BCCAO (*n=20*)	Injection into cisterna magna	01 µg/kg b.wt.	10.92 µg/kg b.wt. (containing 01 µg/kg b.wt. TNF- α)	Inflammatory pathway; Oxidative Stress system	Significant reduction in brain edema ratio as compared to sham group Significant increase in SOD level and decrease in MDA level as compared to sham group Levels of anti-inflammatory cytokines are increased and pro-inflammatory cytokines are decreased as compared to sham group Significantly downregulated the expressions of pro-apoptotic factors as compared to sham group	Significant reduction in brain edema ratio as compared to free TNF-α group Significant increase in SOD level and decrease in MDA level as compared to free TNF-α group Levels of anti-inflammatory cytokines are increased and pro-inflammatory cytokines are decreased as compared to free TNF-α group Significantly downregulated the expressions of pro-apoptotic factors as compared to free TNF-α group	108
10.	Catalase and SOD	PLGA	-	Male Sprague Dawley Rats (*n=3*)	ICA catheter	02 mg/kg b.wt. (tPA)	08 mg/kg b.wt. (Catalase) and 04 mg/kg b.wt. (SOD)	Inflammatory pathway; Oxidative Stress system	Fewer presence of radial glia like neural precursor cells and nestin positive cells after tPA treatment only Large number of caspase positive cells and neutrophils No significant prevention from brain edema post tPA treatment	Widespread distribution of radial glia like neural precursor cells and nestin positive cells Fewer caspase positive cells and neutrophils Inhibited brain edema and protected BBB from ROS	204
11.	Edaravone	PEG-PLA Agonistic Micelles	A_2A_R agonistic agents	Male ICR mice; MCAO (*n=5*)	IV	0.45 μmol	0.45 μmol	BBB permeability	Less drug availability in brain	Up-regulated endothelial monolayer permeability More drug availability in brain compared to other groups Accelerates axonal remodeling post ischemia Improves functional behavior in ischemic stroke model	205
12.	Lycopene	Liposomes	-	Male Sprague Dawley Rats; MCAO (*N=69*)	Intragastric	06 mg/kg b.wt.	06 mg/kg b.wt.	Iron regulating proteins	Reduction in infarct volume Significant reduction in apoptotic cells compared to sham group only Reduced oxidative stress levels compared to sham group Limited suppression of iron regulating proteins Limited suppression of IL-6 release post ischemic insult	Significant reduction in infarct volume compared to free lycopene Significant suppression of apoptosis and oxidative stress Significant suppression of iron regulating proteins like hepcidin and ferroprotein, thus normalizing the iron levels post ischemia Attenuate the release of IL-6	206
13.	Squalenoyl Adenosine (SQAd)	SQAd Nano- assemblies	-	Male Swiss Albino Mice; MCAO (*n=6*)	IV	5.5 mg/kg b.wt.	7.5 or 15 mg/kg b.wt.	Cerebral Micro vessels	Reduction in infarct volume as compared to vehicle group	Significant reduction in infarct volume in ischemic rats as compared to other groups Significant reduction in the apoptotic process in ischemic region Significant improvement in microcirculation	207
14.	Gallic Acid	O-Carboxymethyl Chitosan	-	Male Sprague Dawley Rats; MCAO (*n=8*)	Oral	50 mg/kg b.wt.	50 mg/kg b.wt.	Inflammatory pathway	Significant reduction in infarct volume as compared to control group Reduction in TUNEL-positive cells in comparison to sham group Low anti-inflammatory effects compared to nano group	Best protective effect on infarct size Fewer TUNEL-positive cells post ischemia Significant reduction in TNF-α and IL-1β levels and increase in SOD and catalase levels indicating strong anti-inflammatory effects	99
15.	Osteopontin	Gelatin microspheres	-	Male Sprague Dawley Rats; MCAO (*n=3-6*)	IN	01 µg/rat	01 µg/rat	-	70.22% reduction in infarct size post ischemia Therapeutic effects are seen only if administered within 6 h post MCAO surgery	Enhanced neuroprotective effects of Osteopontin 71.57% reduction in infarct size post ischemia Extension of therapeutic window of IN administered drug to at least 6 h post ischemia	208
16.	NR2B9c Peptide	PEG-PLGA	Wheat Germ Agglutinin	Rats; MCAO (*n=7-8*)	IN	0.3 mg/kg b.wt.	0.3 mg/kg b.wt.	NMDAR	Lower drug availability in brain No significant effect on infarct size and neurological deficit score	Greater bioaccumulation in damaged region due to MCAO Significant reduction in infarct size and neurological deficit scores	209
17.	17β-Estradiol	Gelatin	-	Male C57BL/6J Mice; MCAO (*n=4*)	IN			-	No significant effect on infarct volume	5.24-fold increase in estradiol content in brain 54.3% reduction in infarct volume on 100 ng dose	210

**Table 3:** Antioxidant nanoparticles targeting mitochondrial oxidative stress in ischemic stroke.

S. No.	Therapeutic Loading Molecule	Nanocarriers Used	Mode of Action	References
1.	Cerium Oxide/ Ceria	Polyethylene glycol; TPP	Mimic SOD Activity; Anti-inflammatory effects	89, 94, 17, 97, 98
2.	Silica	Silica NPs		
3.	Iron Oxide	Iron oxide NPs		
4.	Platinum	PtNPs		
5.	Selenium	Selenium NPs		
6.	Free Radical Scavengers	Polyanhydrides; Chitosan; TPP; Polyethylene glycol	Scavenging ROS; Inhibiting Oxidative Stress Damage	89
7.	Gallic Acid	Chitosan	Reduce Oxidative Stress	99
8.	Resveratrol	Solid-Lipid Nanoparticles; Polymer nanoparticles	Enhance mitochondrial function; Reduce LDH and MDA content release	100
9.	Curcumin	DQAsomes; PLGA-PEG; Solid-Lipid nanoparticles	Antioxidant effect	90, 101, 102
10.	Quercetin	PLGA	Scavenging ROS; Inhibiting Oxidative Stress Damage	103, 104
11.	TNF-α	Polyethylene glycol	Attenuate oxidative stress and inflammatory response post IS injury	108
12.	Activated SOD enzyme	PLGA; Liposomes	ROS scavenging; Anti-inflammatory response	109

**Table 4:** List of the molecular moiety and benefits of different nanocarriers.

S. No.	Nanocarriers	Molecular Moiety	Benefits		References
**Organic Nanocarriers**					
1.	Polymeric	PLA, PGA, PLGA, polycaprolactone (PCL) and polyethylene glycol (PEG), chitosan, polysaccharide, gelatin, starch		High biocompatibility, nontoxic by-products within the body and good sustained-release profiles	17, 131
2.	Dendrimers	Polyamidoamine (PAA), Polypropylenimine, Polyaryl ether (PAE)		Capable of encapsulating hydrophilic as well as hydrophobic molecules; Capable to cross various cell membranes or biological barriers, including the BBB through endocytosis	130, 132
3.	Nanogels	Water soluble and Cross-linked polymers like PEG		Greater surface area, unique softness and better drug loading capacity	133
4.	Micelles	L,D-lactine polycaprolactone, PEG		Improved drug stability and bioavailability	86, 134
5.	Liposomes	Spherical vesicles comprising of aqueous core surrounded with single or multiple amphiphilic lipid bilayers		Capable of encapsulating hydrophilic as well as hydrophobic molecules; efficiently delivery of therapeutic molecules, including drugs, vaccines, enzymes, proteins, and nucleic acids, and imaging agents for diagnostics by transcytosis, endocytosis and BBB disruption	131, 135
6.	SLNP	Colloidal nanocarriers comprising of surfactant-stabilized triglycerides, monoglycerides, complex glyceride mixtures, or waxes, hard fats		Enhanced entrapment efficiency for hydrophobic drugs; possess the advantages of both liposomes and polymeric nanoparticles; high physical stability, bioavailability, biocompatibility, drug protection, strict control of release, ease of preparation, efficient tolerance, and biodegradability without generating toxic by-products	136, 137
**Inorganic Nanocarriers**					
1.	Fullerenes	Carbon-based nanomaterial structure; an allotrope of carbon formed as C60 and C70		Antioxidant nature; effective in crossing the BBB when hybridized with a biologically active moiety; prevent disruption and leakage of mitochondrial membrane	132, 139, 142
2.	Graphene	2D single layer of strongly packed carbon atoms; Hydroxyl, epoxyl and carboxyl groups modify the graphene to provide graphene oxide		Antioxidant responses; Anti-inflammatory responses; high drug target specificity; high drug efficiency	148, 150, 153
3.	Carbon Nanotubes	Cylindrical shaped carbon-based nanostructures		Increased surface area; High penetration power; Promotes neuronal activity, network communication and synaptic formation	154
**Biological Vectors as Nanocarriers**					
1.	Viral Vectors	Retrovirus vectors, adenovirus vectors, lentivirus vectors, herpes simplex virus type 1, and adeno-associated virus vectors		Delivers a normal copy of a defective gene and subsequently reduce the harmful functions, thus fighting disease pathology	131, 158
2.	Extracellular Vesicles	Heterogenous cell-derived membrane structures; Exosomes and micro-vesicles		Easily cross BBB by adsorptive/receptor-mediated transcytosis; efficient drug delivery	161, 162
					

**Table 5:** Prominent Routes of Drug Administration for the treatment of neurological disorders.

S. No.	Route	Merits	Limitations
1.	Oral	Easy to administer	Irritation and nausea in some cases
		Easy absorption along the whole length of gastro-intestinal tract	First-pass effect
		Suitable for administration of high doses	Low drug stability due to effects of gastric juices
			Slow release of drug into the circulation
			Not suitable for drugs to be delivered to different brain regions
2.	Inhalation/Intranasal	Large surface area for absorption of drug into the circulation	Dose regulation and precision is not achieved easily
		Direct drug delivery into the circulation	Only suitable for administration of low molecular weight drugs
		Complete drug stability	Inconvenient for drug administration
		Bypass first-pass effect	
		Bypass BBB, thus suitable for direct brain delivery of drugs	
3.	Intravenous	Direct delivery of drug into the circulation	Risk of infection and vessel puncture
		Complete drug bioavailability in blood	Technical assistance required
		Complete drug stability	Inconvenient and comparatively unsafe
		No first-pass effect	
		Suitable for drugs to treat various CNS disorders	
4.	Rectal	Bypass first-pass effect	Irregular absorption
		Steady release of drug	Inconvenient
			Not suitable for drugs to treat several CNS disorders
			Slow absorption rate
5.	Intracerebral	Direct drug delivery to desired brain region	High technical expertise required
		Maximum drug availability in affected brain region	Unsuitable for regular drug administration
		Rapid drug release and effect	High risk of infection
